# Exposure to Emerging Contaminants Chlorinated Paraffins in PM_2.5_ and Sleep Disorders in Youth: Body Weight as a Mediator

**DOI:** 10.3390/toxics14070607

**Published:** 2026-07-11

**Authors:** Wan-Ting He, Jing-Wen Huang, Xuan Liu, Muhammad Amjad, Yan-Xu Chen, Kun Zhao, Yun-Ting Zhang, Chu Chu, Yang Zhou, Li-Zi Lin, Wen-Wen Bao, Haseeb Tufail Moryani, Ru-Qing Liu, Xi-Fei Yang, Pei-Pei Wang, Guang-Hui Dong

**Affiliations:** 1 Joint International Research Laboratory of Environment and Health, Ministry of Education, Guangdong Provincial Engineering Technology Research Center of Environmental Pollution and Health Risk Assessment, Department of Occupational and Environmental Health, School of Public Health, Sun Yat-sen University, Guangzhou 510080, China; hewt36@mail2.sysu.edu.cn (W.-T.H.); huangjw278@mail.sysu.edu.cn (J.-W.H.); liux776@mail2.sysu.edu.cn (X.L.); muhammad7@mail.sysu.edu.cn (M.A.); chenyx833@mail2.sysu.edu.cn (Y.-X.C.); zhaok55@mail2.sysu.edu.cn (K.Z.); zhangyt47@mail2.sysu.edu.cn (Y.-T.Z.); chuch3@mail3.sysu.edu.cn (C.C.); zhouyang5@mail.sysu.edu.cn (Y.Z.); linlz@mail.sysu.edu.cn (L.-Z.L.); baoww5@mail.sysu.edu.cn (W.-W.B.); haseeb.moriani@gmail.com (H.T.M.); liurq@mail.sysu.edu.cn (R.-Q.L.); donggh5@mail.sysu.edu.cn (G.-H.D.); 2 Shenzhen Key Laboratory of Modern Toxicology, Shenzhen Medical Key Discipline of Health Toxicology, Shenzhen Center for Disease Control and Prevention, No. 8, Longyuan Road, Nanshan District, Shenzhen 518055, China; 3 Sleep Medicine Department, Sanya Central Hospital (The Third People’s Hospital of Hainan Province), No. 1154, Jiefang Fourth Road, Sanya 572000, China

**Keywords:** chlorinated paraffins, sleep disorder, mediation analysis, body weight, fine particle matter component

## Abstract

Chlorinated paraffins (CPs) are emerging contaminants with potential risks to the environment and human health, but their link between fine particulate matter-bound chlorinated paraffins (PM_2.5_-bound CPs) and the risk of sleep disorders has not been reported. This large-scale, population-based study included 122,965 valid questionnaires from school-aged children in the Pearl River Delta region of China. Generalized linear mixed models, restricted cubic splines, weighted quantile sum regression and Quantile g-computation models were used to evaluate the individual and combined effects of PM_2.5_-bound CPs on sleep disorders. Mediation analysis assessed the potential role of body weight. Our findings showed that an interquartile range (IQR) increase in PM_2.5_-bound ∑CPs was linked to a higher risk of sleep disorders, and odds ratio ranging from 1.08 to 3.20. Short-chain chlorinated paraffins (SCCPs) were identified as key contributors of CPs and might interfere with important metabolic pathways. Further analysis indicated that body weight statistically accounted for part of the observed association between exposure to CPs and sleep disorders, with the proportion mediated ranging from 3.40% to 35.67%. Stratified analyses suggested stronger associations among girls and children under 12 years. These findings support prioritizing CPs management strategies to reduce their negative impact among children and adolescents.

## 1. Introduction

Environmental exposures have increasingly been recognized as important determinants of human health, including emerging impacts on sleep health. Particulate matter, a complex mixture of chemical constituents, has been linked to a range of adverse health outcomes [1]. Growing evidence suggests that fine particulate matter (PM_2.5_) exposure may also impair sleep quality and increase the risk of sleep disorders, particularly among children and adolescents [2]. However, the toxicity of PM_2.5_ is largely driven by its chemical composition, and current knowledge regarding the effects of specific PM_2.5_-bound components on sleep health remains limited.

Chlorinated paraffins (CPs) are a class of emerging contaminants and environmentally persistent industrial chemicals. Owing to their hydrophobicity and particle-binding properties, CPs can readily adsorb onto PM_2.5_ particles, facilitating long-range atmospheric transport and contributing to widespread environmental contamination [3,4]. These compounds are classified by carbon chain length into short- (SCCPs), medium (MCCPs), and long-chain (LCCPs) categories. According to emission modeling [5], Guangdong is one of the predominantly primary regions responsible for SCCP releases, highlighting their significant role in environmental emissions. With global regulatory efforts to phase out SCCPs, the use of MCCPs and LCCPs has increased substantially, changing human exposure profiles and raising concerns about their health effects [6]. Toxicological studies show that CPs have neurotoxic, immunotoxic, and developmental and reproductive impairments [7,8,9,10]. For example, SCCPs have been demonstrated to impair zebrafish locomotor activity and disrupt blood–brain barrier integrity, while short-term exposure in mice caused systemic insulin resistance and macrophage-driven inflammation [11,12]. Similarly, MCCPs have also been reported to disturb cerebral lipid homeostasis and exacerbate neurological injury [13]. In addition, research further indicates that SCCPs adsorbed to particulate matter interfere with cellular metabolism and induce oxidative stress through complex toxic interactions [14]. Despite these advancements, epidemiological evidence linking PM_2.5_-bound CPs exposure to specific health outcomes remains scarce, particularly regarding sleep health in pediatric populations.

Sleep disorders, which include inadequate sleep duration, abnormal nighttime behaviors, and disruptions in circadian rhythm, are an underrecognized and growing public health problem [15,16,17]. Globally, these conditions affect about 20% of children and adolescents, with prevalence in China nearing 40% [18,19]. The negative effects of irregular sleep patterns on the mental and social health of school-aged children can influence their future development and may increase the long-term risks of hypertension, type 2 diabetes, and cardiovascular diseases [20,21,22,23,24]. Identifying modifiable risk factors, such as excess weight and exposure to environmental pollutants, and understanding their underlying mechanisms could provide vital evidence for developing targeted interventions to improve sleep outcomes in vulnerable populations.

Among these factors, body weight may contribute to the observed association between environmental exposures and sleep outcomes. Obesity is closely associated with sleep disturbances and may share common metabolic and inflammatory pathways with environmental toxicants [25,26,27]. However, whether body weight is associated with exposure to PM_2.5_-bound CPs and sleep disorders remains unclear.

To address this knowledge gap, we conducted a large-scale, population-based study in the Pearl River Delta region of China. A total of 131,412 children and adolescents were initially recruited, and 122,965 valid questionnaires were ultimately included in the analysis. This study aimed to evaluate the relationships between PM_2.5_-bound CPs exposure and sleep disorders and their subtypes, using generalized linear mixed models, restricted cubic splines, and weighted quantile sum regression. Mediation analysis and network toxicology were also applied to explore potential biological mechanisms underlying these associations.

## 2. Materials and Methods

### 2.1. Study Participants

Between May 2016 and May 2018, a total of 131,412 participants aged 6–18 years were recruited from primary and secondary schools across six cities in the PRD region of China. The locations and distribution of the 105 sampled schools have been documented in previous studies [28] and are detailed in Supplementary Figure S1 and Table S1. We collected a total of 122,965 valid questionnaires from children and adolescents. The detailed recruitment process for participants is described in Supplementary Text S1. Approval for all procedures was granted by the Ethics Committee of Sun Yat-sen University (2018057). Written informed consent for publication of study data was obtained from the parents or guardians of all participating children.

### 2.2. Sleep Disorders Measurement

The Chinese Sleep Disturbance Scale for Children (SDSC), which has good validity (Cronbach’s alpha = 0.81), was used to assess sleep disorders in participants. Briefly, the SDSC questionnaire includes 26 items related to sleep quality over six months, and higher scores in children indicate a greater risk for sleep problems. All SDSC items were evaluated with a 5-point Likert scale, covering total sleep duration, sleep latency, and other subtypes. The total sleep score on the SDSC ranges from 26 to 130 points, and with specific scoring ranges or different sleep disorder subtypes: sleep initiation and maintenance disorders (DIMS) from 7 to 35, sleep–wake transition disorders from 6 to 30, excessive daytime sleepiness disorders (DOES) from 5 to 25, sleep breathing disorders (SBD) from 3 to 15, disorders of arousal from 3 to 15, and sleep hyperhidrosis (SHY) from 2 to 10. According to guidelines, we also defined shorter sleep duration (<7 h) and longer sleep latency (>45 min) based on the first two items from the SDSC.

### 2.3. Chlorinated Paraffin Analysis

PM_2.5_ samples were collected from the participating schools in 2018 using medium- and high-volume air samplers. PM_2.5_-bound CP exposure was assigned to participants based on the measurements from their corresponding schools. The analysis of PM_2.5_-bound CPs, including SCCPs, MCCPs, and LCCPs compounds, was conducted based on our previously established method [29]. Briefly, sample extracts were purified and analyzed using ultra-performance liquid chromatography coupled with quadrupole time-of-flight mass spectrometry (UPLC-QTOF-MS). Detailed procedures on sample preparation, quality control, and quantification are described in Supplementary Text S2.

### 2.4. Network Toxicology Analysis

#### Screening of Targets, Functional and Pathway Enrichment

The environmental and health risks increase with shorter carbon chain lengths in CPs. Therefore, a network toxicology analysis was conducted to explore the underlying mechanisms of SCCP-induced sleep disorders. Multiple databases, including NetInfer (https://lmmd.ecust.edu.cn/netinfer/; accessed on 15 August 2025), SwissTargetPrediction (https://www.molecular-modelling.ch/swiss-drug-design.html; accessed on 15 August 2025), SuperPred (https://prediction.charite.de/index.php; accessed on 15 August 2025), PharmMapper (https://lilab-ecust.cn/pharmmapper/index.html; accessed on 15 August 2025), and TargetNet (http://targetnet.scbdd.com/home/index/; accessed on 15 August 2025), were used to identify potential targets of SCCPs. The chemical structures and SMILES of SCCPs were drawn using the SwissTargetPrediction database and used as search terms to find relevant gene sets. The target genes of SCCPs were identified by taking the union of the predicted genes from the five databases. Gene Ontology (GO) analysis and Kyoto Encyclopedia of Genes and Genomes (KEGG) analysis were conducted to investigate the biological events of SCCPs. The top 10 terms and 15 pathways were visualized based on a criterion of adjusted *p* value < 0.05.

### 2.5. Statistical Analysis

The concentration level of CPs was obtained by calculating the arithmetic mean of PM_2.5_ sampling data collected during summer and winter. Descriptive statistics summarized the characteristics of the study objects, with categorical variables shown as frequencies and continuous variables as means with standard deviations. The distributions of CPs concentrations were displayed as percentiles. Mann–Whitney U test, and chi-square test were performed to compare different groups. Effect size estimates were additionally reported to complement *p*-values; specifically, Cohen’s d was reported for continuous variables, and Cramer’s V was reported for categorical variables.

#### 2.5.1. Generalized Linear Mixed Model Analyses

To evaluate the association between CPs exposure and the sleep disorder risk, we used generalized linear mixed models (GLMMs) to estimate the percentage change in the odds ratio (OR) of sleep disorders (with a 95% confidence interval, CI) per interquartile range (IQR) increase in CPs. Participants were also divided into four groups based on CPs concentration quartiles for trend testing. The main model adjusted for variables such as sex, age, body mass index (BMI) category, maternal age, cesarean delivery, preterm birth, low birth weight, breastfeeding status, parental education, household income, health insurance, physical activity (PA), secondhand smoke (SHS) exposure, pet ownership, and PM_2.5_ levels. Additionally, city was included as a random effect to address potential regional clustering.

#### 2.5.2. Restricted Cubic Spline Analyses

Restricted cubic splines (RCS) with 3 knots at the 10th, 50th and 90th percentiles of CPs distribution among participants were used to estimate the dose–response relationships between CPs exposure and the risk of sleep disorders, adjusting for the same set of covariates as the main model.

#### 2.5.3. Mediation Analyses

Mediation analyses were performed after adjusting for the same covariates with main model to examine the role of body weight in the relationship between ∑CPs exposure and sleep disorders and their subtypes. BMI was used as a measure of body weight, and the proportions of indirect, direct, total, and mediation effects were calculated. The bootstrap resampling method was employed to determine the significance of the *p* value and the 95% confidence intervals for the mediation effects, including indirect effect (IE), direct effect (DE), total effect (TE), and proportion of mediation (PM).

#### 2.5.4. Mixture Analysis

To evaluate the combined effect of chlorinated paraffin (CP) mixtures, we used Quantile g-computation (qgcomp) models and weighted quantile sum (WQS) regression models. These models evaluated the overall mixture impact on the risk of sleep disorder and its subtypes, while also estimating the relative contribution weights of individual CP components (∑SCCPs, ∑MCCPs, ∑LCCPs). The covariates adjusted for in the WQS and qgcomp models were consistent with the main model. The overall association was represented by the odds ratio (OR) for a simultaneous one-quantile increase in all CP exposures.

#### 2.5.5. Stratified Analyses and Sensitivity Analysis

Stratified analyses were used to evaluate how sex and age influence the relationship between ∑CPs exposure and the risk of sleep disorders and their subtypes in populations with different characteristics. Participants were grouped based on sex (boys and girls) and age (≤12 years and >12 years). Sensitivity analysis was conducted to test the stability of the results. First, ∑CP exposure and sleep score were standardized using z-score transformation, and the models were refitted with effect estimates expressed per one-standard-deviation increase in exposure. Second, ∑CP exposures were categorized into quartiles, with the lowest quartile used as the reference group. Third, the analyses were repeated after excluding participants who were not breastfed, those with low birth weight, and those with preterm birth. All sensitivity analyses were adjusted for the same covariates as the main models.

All statistical analyses were performed using R (version 4.2.2), *p* < 0.05 was considered statistically significant.

## 3. Results

### 3.1. Characteristic Features of Study Subjects

The baseline characteristics of 122,965 participants are shown in Table 1. The prevalence of sleep disorder is 4.8% (*n* = 5909). Subjects without sleep disorder had an average age of 11.74 (2.94) years, and were 52.9% (*n* = 61,971) boys. Subjects with sleep disorder had an average age of 11.88 (3.08) years, and were 54.3% (*n* = 3211) boys. Compared to the group without sleep disorder, those with sleep disorder had significantly higher proportions of overweight/obesity (20.8%), high maternal age (16.8%), preterm birth (7.8%), low birth weight (9.7%), less breastfeeding (53.0%), higher parental education levels (47.5%), lower family income (23.0%), less physical activity (26.7%), secondhand smoke exposure (41.8%) and pet ownership (25.4%). Additionally, compared to participants without sleep disorder, those with sleep disorder had higher median concentrations of PM_2.5_ (31.88 µg/m^3^ vs. 32.75 µg/m^3^), ∑SCCP (12.75 ng/m^3^ vs. 13.50 ng/m^3^), ∑MCCP (14.16 ng/m^3^ vs. 14.26 ng/m^3^), and ∑CP (28.39 ng/m^3^ vs. 29.42 ng/m^3^) (Table 1).

### 3.2. Associations of Individual ∑CPs Exposures with Sleep Disorder and Its Subtypes

All participants were divided into different groups based on their level of exposure to ∑CPs. In Table S2, the high ∑CPs exposure group had higher sleep disturbance scores (52.79 vs. 50.88) and higher incidences of sleep disorder (5.2% vs. 4.4%), compared to the low ∑CPs exposure group. The results of the GLMM analysis are shown in Table 2. After adjusting for covariates, the analysis revealed that ∑CPs exposure concentration by an interquartile range was linked to a higher likelihood of sleep disorder, with an odds ratio of 1.18 (95% CI: 1.15, 1.21). There was a notable positive link between an IQR rise in exposure to ∑SCCPs, ∑MCCPs, and ∑LCCPs and the likelihood of sleep disorders in the fully adjusted model, with odds ratios (ORs) ranging from 1.06 (95% CI: 1.04, 1.09) to 1.16 (95% CI: 1.13, 1.19). Similar associations were seen between CPs exposure and higher odds ratios for various subtypes, with OR and ranging from 1.08 (95% CI: 1.05, 1.11) to 3.20 (95% CI: 3.13, 3.26). Consistent findings were observed between exposure and the risk of various subtypes. Furthermore, the associations between ∑CPs in PM_2.5_ and sleep scores are detailed in Table 2. We found that sleep disturbance and subtype scores were positively associated with ∑CP exposure concentrations, with OR ranging from 1.25 (95% CI: 1.19, 1.31) to 1.88 (95% CI: 1.86, 1.99). Similar associations were also observed between CPs with different carbon chain lengths and sleep disturbance and dimension scores, with *β* values ranging from 0.42 (95% CI: 0.38, 0.47) to 1.66 (95% CI: 1.60, 1.72).

To further evaluate the shape of the associations, dose–response relationships were analyzed using restricted cubic splines (RCS) for concentrations of CPs and the risks of overall sleep disorder and its subtypes. As shown in Figure S2, ∑CP, ∑SCCP, and ∑MCCP showed similar monotonically increasing dose–response trends regarding the risk of sleep disorder and its subtypes, including DIMS, DA, SWTD, DOES, shorter sleep duration, and prolonged sleep latency. In contrast, the dose–response relationship between ∑LCCP and the risk of sleep disorders demonstrated that ∑LCCP had a J-shaped dose–response curve with the subtypes Sleep disorder, DIMS, SBD, SWTD, and DOES, while an inverted U-shaped dose–response curve was observed with SHY subtype.

### 3.3. Associations of ∑CPs Mixture Effect with Sleep Disorder and Its Subtypes

**Figure 1:**
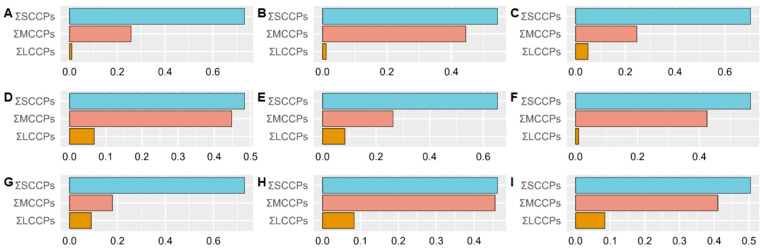
Estimated weights of individual CPs in the associations with the risk of sleep disorder and subtypes with WQS model. (**A**–**I**) indicate sleep disorder, DA, DOES, SBD, SWTD, SHY, DIMS, shorter sleep duration and long sleep latency.

WQS regression models and qgcomp models were used to assess the joint effects of CPs mixtures and the effect of each ∑CPs component’s concentration on the likelihood of sleep disorders and their subtypes. WQS regression analysis revealed that a one-quartile elevation in CPs mixture concentration was positively related to the likelihood of sleep disorders. (ORs range: 1.17–3.61), as detailed in Table S3. The qgcomp models showed that each one-quantile increase in the CPs mixture was associated with higher risk of sleep disorders, with RRs ranging from 1.12 to 2.98 (Table S4). Additionally, both weight analyses suggested that the association was mainly influenced by ∑SCCPs (Figure 1 and Figure S3).

### 3.4. Network Toxicology Analysis of SCCP-Related Genes and Pathways

SCCP was identified as the main factor behind the joint effects of CPs. To better understand its toxicological mechanisms, additional toxicology analyses were performed. As shown in Figure S4A, key genes related to SCCP were identified. Then, Gene Ontology analysis showed significant enrichment in key biological processes such as G protein-coupled receptor signaling, response to peptide hormones and bacterial molecules, and regulation of calcium and MAPK signaling pathways (Figure S4B). Further pathway enrichment analysis indicated important involvement of biological pathways related to neural signaling and metabolic regulation, including neuroactive ligand-receptor interaction, calcium signaling, cAMP, PI3K-Akt, MAPK, hormone, and lipid and atherosclerosis signaling (Figure S4C).

### 3.5. Potential Role of Body Weight in the CPs-Sleep Disorder Association

**Figure 2:**
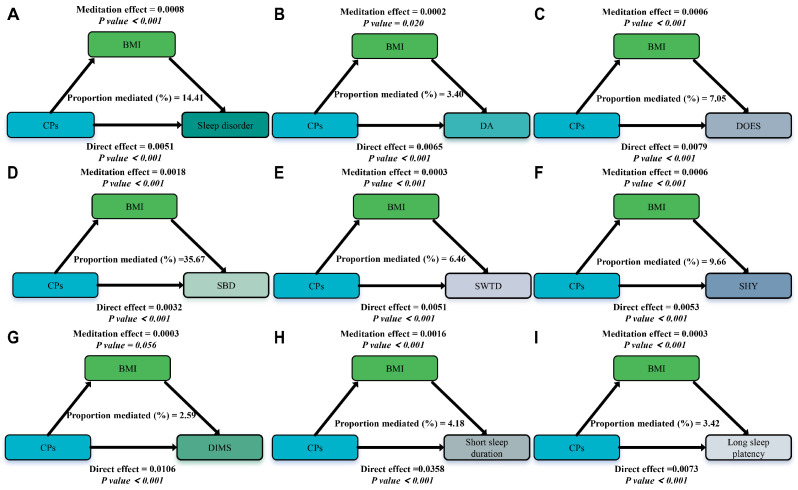
Effects of ∑CPs exposure on sleep disorder (**A**) and its subtypes (**B**–**I**) via BMI. Mediation analysis models were adjusted for sex, age, physical activity, low birth weight, premature birth, caesarean, breastfeeding, parental education, maternal age, insurance, family income class, secondhand smoking exposure and pet ownership and PM_2.5_ concentration.

Building on network toxicology analysis and previous evidence indicating that CPs disrupt metabolic homeostasis, we conducted mediation analysis to assess the potential mediating role of body weight in this pathway. Correlation analysis showed that BMI was significantly correlated with CPs concentration and sleep disorder and its subtypes (Figure S5 and Tables S5 and S6). As shown in Figure 2A–I and Table S7, further mediation analyses revealed that BMI may contribute to the observed association between ∑CPs exposure and the risk of sleep disorder (OR = 1.0009, 95% CI: 1.0006–1.0011; *p*  <  0.001), with mediation percentages of 14.41%. Furthermore, mediation analysis demonstrated that BMI also contributed to the observed association between ∑CPs exposure and multiple sleep disorder subtypes, including SBD, DA, DOES, SHY, and SWTD, with proportions mediated ranging from 3.40% to 35.67%. Notably, the SBD and SHY subtypes showed the highest mediation proportions, with BMI accounting for 35.67% (OR: 1.0018, 95% CI: 1.0015–1.0020; *p* <  0.001) and 9.66% (OR: 1.0006, 95% CI: 1.0002–1.0009; *p*  <  0.001), respectively. We also explored the mediating effect of BMI on the associations between ∑CPs exposure with the sleep duration and sleep latency. Similarly, BMI significantly mediated the relationship between ∑CPs exposure and shorter sleep duration (Proportion = 4.18%; OR: 1.0016, 95% CI: 1.0014–1.0017) and longer sleep latency (Proportion = 3.42%; OR: 1.0003, 95% CI: 1.0001–1.0014).

### 3.6. Stratified Analyses and Sensitivity Analyses

**Figure 3:**
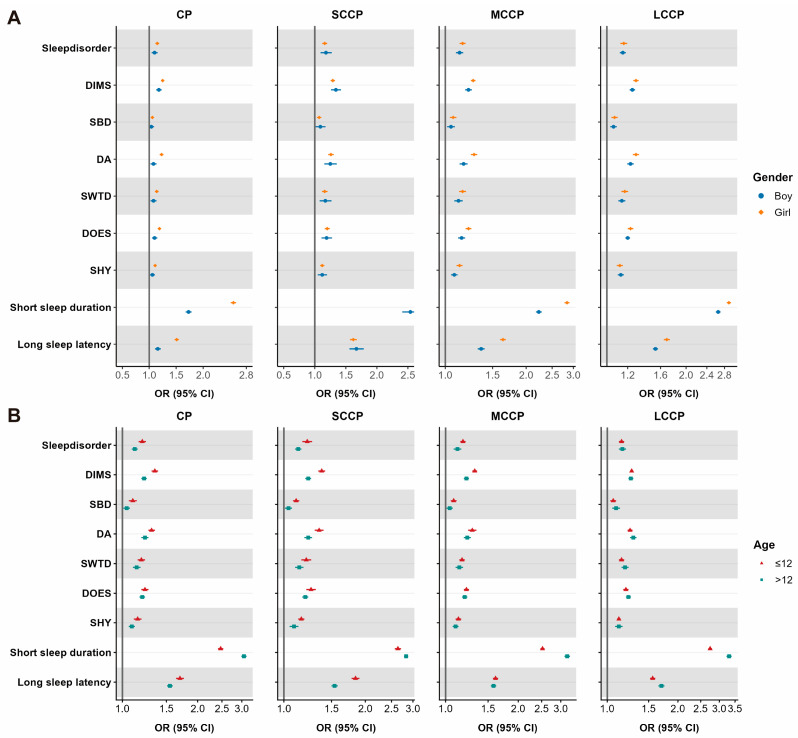
Stratified analyses by sex (**A**) and age (**B**) revealed significant modification effects. Adjusted for BMI class, physical activity, low birth weight, premature birth, caesarean, breastfeeding, parental education, maternal age, insurance, family income class, secondhand smoking exposure, pet ownership and PM_2.5_ concentration.

Stratified analyses by sex and age showed significant modification effects (Figure 3; Tables S8 and S9). Sex-specific patterns emerged, with girls having significantly higher odds of sleep disorder associated with ∑CPs exposure compared to boys (OR: 1.16, 95% CI: 1.13–1.19 vs. OR: 1.13, 95% CI: 1.06–1.17; *p _for interaction_* < 0.05), indicating greater female susceptibility to these environmental exposures. Age also significantly influenced the association, as children under 12 years had increased risks of overall sleep disorders following ∑CPs exposure (OR: 1.20, 95% CI: 1.16–1.24 vs. OR: 1.12, 95% CI: 1.09–1.16; *p _for interaction_* < 0.05), while those over 12 years showed higher risks specifically for shortened sleep duration (OR: 3.06, 95% CI: 2.99–3.13 vs. OR: 2.47, 95% CI: 2.41–2.53; *p _for interaction_* < 0.05).

Several sensitivity analyses were additionally conducted. First, after standardizing ∑CP exposures and sleep score by z-score transformation, the direction of the associations was generally consistent with the main analyses, although some estimates were attenuated (Table S10). Second, dividing the study population by CPs quartiles provided further support for the robustness of the association (Tables S11 and S12). Results from sensitivity analyses excluding children born preterm, with low birth weight, or not exclusively breastfed remained consistent. These analyses repeatedly showed a significant positive link between ∑CPs exposure and sleep disorders and their subtypes (Table S13–S15), further reinforcing the primary study conclusions.

## 4. Discussion

This study provides novel epidemiological evidence of positive associations between CP exposure and sleep disorders and their subtypes among children and adolescents, with ∑SCCPs identified as the main risk factors. Network toxicology indicates that CPs may disrupt metabolic balance and nervous system function. Additionally, mediation analysis shows that body weight partially mediates the positive links between CPs exposure and the risk of sleep disorders and specific subtypes. We also found differences in susceptibility, with girls and children under 12 years old showing greater vulnerability to ∑CPs exposure. These findings offer valuable insights for developing targeted CPs management strategies to reduce their negative effects on sleep health in the pediatric population.

Few studies have examined the effects of PM_2.5_ component exposure on sleep disorders in children and adolescents, and none have assessed the effects of CPs on sleep disorders. Previous research has found that exposure to air pollutants increases the odds of sleep disorders in children, especially those associated with harmful PM_2.5_ components like organic matter and black carbon [30,31,32]. At the same time, evidence also confirms that exposure to persistent organic pollutants may shorten sleep duration and impair sleep quality. PFAS, including PFDA, PFOA, and PFHxS, have been associated with shorter sleep duration, while PFOS has been linked to greater sleep disturbance and sleep-related impairment, potentially involving metabolic and immune function-related proteins [33]. Additionally, the HOME Study reported that gestational exposure to PBDE-99, PBDE-47, and ΣPBDEs was associated with greater sleep irregularity and sleep disruption in children aged 2–8 years, but not with sleep duration [34]. The current study is the first to provide new evidence that CPs adsorbed to PM_2.5_ play a significant role in increasing the risk of sleep disorders and their subtypes in children and adolescents.

Body weight is a significant reversible etiological risk factor for many health outcomes [35]. According to Zhang et al., prolonged exposure to air pollution, particularly certain particulate matter components, is linked to weight gain and a higher rate of obesity among children and adolescents [36]. Moreover, a national analysis indicated that exposure to PM_2.5_ and its constituents, particularly black carbon (BC), was significantly linked to an elevated risk of metabolic syndrome [37]. Consistent with these findings, our research supports that body weight was positively associated with sleep disorders (OR:1.04, 95% CI: 1.03, 1.06) and other subtypes (OR ranging from 1.02 to 1.16). Furthermore, the comprehensive relationship between obesity and sleep disorders has been revealed. Obesity is an independent factor for the development of difficulties in maintaining sleep, excessive daytime sleepiness, insomnia and sleep-related breathing disorders [38,39,40,41,42]. Additionally, our research results revealed that body weight mediates the association between ∑CP exposure and the risk of sleep disorders and their subtypes, especially SBD and SHY. These findings suggest that weight control may serve as a modifiable factor in CP-induced sleep disorders, providing a novel management strategy for addressing sleep disorders caused by CP exposure.

Our study also indicates that the pattern of how sex and age influence the effect of CPs exposure on sleep disorders is important. Gender differences may be observed in the responses to environmental pollutant exposure [43]. Fluctuations in hormonal rhythms during female adolescence and greater body fat are significant factors that modulate sleep disorders [44,45]. Specifically, environmental pollutants can disrupt pubertal development by interfering with the synthesis of key hormones like DHEA and estradiol [44]. Consistent with this, we found that girls were more vulnerable to PM_2.5_-bound CPs and had a higher sleep disorder risk. Additionally, compared with older children, younger children were at a significantly higher risk of sleep problems following such exposure. The reason might be that younger children consume more relative to their body weight and frequently engage in hand-to-mouth behavior, resulting in more exposure to pollutants [46,47]. Moreover, the organ functions of young children are still developing, with weaker barrier defenses against chemical pollutants, making this age group more susceptible to exposure during this period. Exposure to pollutants such as PM_2.5_ and persistent organic pollutants is more likely to interfere with normal developmental processes [48,49,50,51].

The potential mechanisms connecting PM_2.5_-bound CPs, obesity, and sleep disorders may involve a complex physiological cascade. As lipophilic compounds, CPs build up in adipose tissue, where they disrupt lipid metabolism and trigger chronic low-grade inflammation, which are key processes in developing obesity [12,13]. This metabolic inflammation, marked by increased pro-inflammatory cytokines, can further affect hypothalamic function and neuroendocrine regulation, impacting sleep–wake cycles [52]. CP-induced oxidative stress and mitochondrial dysfunction in neural tissues may also interfere with the production of sleep-related neurotransmitters and circadian rhythm regulation [12]. Recent research indicates that CP exposure changes gut microbiota composition, potentially contributing to systemic inflammation and sleep disturbances through the gut–brain axis [53,54]. Altogether, these interconnected pathways, including metabolic dysregulation, chronic inflammation, neuroendocrine disruption, and microbiota imbalance, provide a plausible explanation for the link between CP exposure and sleep disorders, with obesity serving as both a mediator and an amplifier in this complex relationship.

Our study has several notable strengths. First, it is the first to suggest that body weight may contribute to the association between CP exposure and the risk of sleep disorders, filling an important gap in the current literature. Second, using validated scales to thoroughly assess sleep quality in children and adolescents improves the reliability of the findings. Third, we identified younger children and girls as susceptible subgroups for CP-related sleep issues, providing a basis for targeted risk assessment. Despite these strengths, several limitations should be acknowledged. First, PM_2.5_-bound CP exposure was assigned in 2018, whereas participant information was collected from 2016 to 2018; therefore, temporal variation in exposure during the study period could not be fully captured, although school locations remained stable. Second, although school-level measurements may partly reflect individual ambient exposure because of China’s nearby enrollment policy and regular outdoor activities during school breaks, they may not capture household-level exposures, indoor gym exposures, commuting patterns, or other individual-level factors, leading to potential exposure misclassification. Third, the cross-sectional design constrains and limits causal conclusions, while regional sampling may restrict how broadly the findings apply. Fourth, the lack of longitudinal data hampers the ability to study long-term exposure effects. Last, several environmental and behavioral factors, including indoor exposures, traffic-related noise, nighttime light exposure, screen time, and other determinants of sleep quality, as well as co-exposures to pesticides, PAHs, and other particle-bound pollutants, were not directly assessed, which may have resulted in residual confounding. Altogether, these interconnected pathways, including metabolic dysregulation, chronic inflammation, neuroendocrine disruption, and microbiota imbalance, may provide a plausible explanation for the association between CP exposure and sleep disorders, with obesity potentially contributing to or modifying this complex relationship.

## 5. Conclusions

PM_2.5_-bound CPs are positively associated with the risk of sleep disorders among children and adolescents in the Pearl River Delta region, with SCCPs identified as the primary contributors. Body weight may statistically explain the association between PM_2.5_-bound CP exposure and sleep-related outcomes. Girls and children under 12 years of age appeared to be more vulnerable. These findings highlight the importance of considering CPs in health risk assessments and prevention strategies. Future longitudinal and mechanistic studies are needed to confirm these associations and clarify the underlying biological mechanisms.

## Figures and Tables

**Table 1:** Demographic characteristics of current study participants stratified by sleep disorder.

Variables	Without Sleep Disorder *n* = 117,056	With Sleep Disorder *n* = 5909	*p* Value	Effect Size
Age, y, mean (SD)	11.74 (2.94)	11.88 (3.08)	<0.001	−0.046
Boys, *n* (%)	61,971 (52.9)	3211 (54.3)	<0.001	0.005
Overweight/obesity, *n* (%)	20,449 (17.5)	1231 (20.8)	<0.001	0.019
Maternal age, *n* (%)			0.016	0.007
≤25	39,875 (34.1)	1908 (32.3)		
25 to 30	58,421 (49.9)	3011 (51.0)		
>30	18,760 (16.0)	990 (16.8)		
Caesarean, *n* (%)	41,045 (35.1)	2097 (35.5)	0.514	0.000
Preterm birth, *n* (%)	5940 (5.1)	461 (7.8)	<0.001	0.026
Low birth weight, *n* (%)	5860 (5.0)	571 (9.7)	<0.001	0.045
Breastfeeding, *n* (%)	68,890 (58.9)	3134 (53.0)	<0.001	0.025
Parental education, high, *n* (%)	52,108 (44.5)	2808 (47.5)	<0.001	0.013
Family income, yuan, *n* (%)			<0.001	0.012
≤10,000	24,483 (20.9)	1360 (23.0)		
10,001~30,000	19,825 (16.9)	1024 (17.3)		
30,001~100,000	38,248 (32.7)	1802 (30.5)		
>100,000	34,500 (29.5)	1723 (29.2)		
Insurance, *n* (%)	95,990 (82.0)	4770 (80.7)	0.013	0.007
Active physical activity, *n* (%)	36,208 (30.9)	1579 (26.7)	<0.001	0.019
Secondhand smoke exposure, *n* (%)	40,258 (34.4)	2471 (41.8)	<0.001	0.033
Pet ownership, *n* (%)	22,916 (19.6)	1501 (25.4)	<0.001	0.031
PM_2.5_, median (IQR), µg/m^3^	31.88 (30.48, 35.98)	32.75 (30.82, 36.45)	<0.001	−0.161
∑SCCP, median (IQR), ng/m^3^	12.75 (9.08, 17.09)	13.50 (9.29, 24.16)	<0.001	
∑MCCP, median (IQR), ng/m^3^	14.16 (10.71, 18.56)	14.26 (10.95, 23.22)	<0.001	−0.172
∑LCCP, median (IQR), ng/m^3^	1.35 (0.84, 2.07)	1.28 (0.78, 1.81)	0.007	−0.148
∑CP, median (IQR), ng/m^3^	28.39 (22.16, 38.44)	29.42 (23.15, 49.38)	<0.001	−0.044

**Table 2:** Association between ∑CPs exposures and the risk of sleep disorder and subtypes among all participants in the logistic model.

Variables	∑CPs	∑SCCPs	∑MCCPs	∑LCCPs
Categorical variables (OR, 95% CI)				
Sleep disorder	**1.18** **(1.15, 1.21)**	**1.16** **(1.13, 1.19)**	**1.13** **(1.10, 1.16)**	**1.06** **(1.04, 1.09)**
DIMS	**1.32** **(1.29, 1.35)**	**1.27** **(1.25, 1.30)**	**1.24** **(1.21, 1.26)**	**1.11** **(1.09, 1.13)**
SBD	**1.08** **(1.05, 1.11)**	**1.07** **(1.04, 1.10)**	**1.06** **(1.03, 1.09)**	**1.03** **(1.01, 1.05)**
DA	**1.35** **(1.31, 1.39)**	**1.26** **(1.23, 1.30)**	**1.28** **(1.24, 1.31)**	**1.12** **(1.10, 1.14)**
SWTD	**1.19** **(1.16, 1.23)**	**1.16** **(1.13, 1.19)**	**1.14** **(1.11, 1.18)**	**1.08** **(1.06, 1.10)**
DOES	**1.26** **(1.23, 1.29)**	**1.21** **(1.18, 1.24)**	**1.21** **(1.18, 1.24)**	**1.09** **(1.07, 1.11)**
SHY	**1.14** **(1.12, 1.17)**	**1.12** **(1.10, 1.15)**	**1.12** **(1.09, 1.14)**	**1.02** **(1.01, 1.04)**
Short sleep duration	**3.20** **(3.13, 3.26)**	**2.77** **(2.72, 2.83)**	**2.28** **(2.24, 2.32)**	**1.36** **(1.34, 1.37)**
Long sleep latency	**1.85** **(1.80, 1.90)**	**1.60** **(1.56, 1.64)**	**1.70** **(1.66, 1.74)**	**1.28** **(1.25, 1.30)**
Continuous variables (β, 95% CI)				
Total t-score	**1.32** **(1.26, 1.39)**	**1.15** **(1.09, 1.21)**	**1.02** **(0.96, 1.08)**	**0.42** **(0.37, 0.47)**
DIMS t-score	**1.66** **(1.59, 1.73)**	**1.41** **(1.35, 1.47)**	**1.29** **(1.23, 1.35)**	**0.59** **(0.54, 0.64)**
SBD t-score	**1.25** **(1.19, 1.31)**	**1.07** **(1.01, 1.13)**	**0.95** **(0.90, 1.01)**	**0.47** **(0.43, 0.51)**
DA t-score	**1.36** **(1.30, 1.42)**	**1.14** **(1.08, 1.20)**	**1.07** **(1.02, 1.12)**	**0.48** **(0.44, 0.52)**
SWTD t-score	**1.31** **(1.24, 1.37)**	**1.13** **(1.07, 1.19)**	**1.01** **(0.95, 1.07)**	**0.43** **(0.38, 0.47)**
DOES t-score	**1.26** **(1.20, 1.33)**	**1.10** **(1.05, 1.16)**	**0.96** **(0.90, 1.02)**	**0.42** **(0.38, 0.47)**
SHY t-score	**1.92** **(1.86, 1.99)**	**1.66** **(1.60, 1.72)**	**1.50** **(1.44, 1.55)**	**0.60** **(0.55, 0.65)**

Model: Adjusted for sex, age, BMI class, physical activity, low birth weight, premature birth, caesarean, breastfeeding, parental education, maternal age, insurance, family income class, second hand smoking exposure and pet ownership; Bold: *p* < 0.05.

## Data Availability

The raw data supporting the conclusions of this article will be made available by the authors on request.

## Supplementary Materials

The following supporting information can be downloaded at: https://www.mdpi.com/article/10.3390/toxics14070607/s1. All supplemental data in Supplementary File, including Text S1. Study Recruitment Details; Figure S1. Schools and sampling site in the Pearl River Delta that were included in this study; Table S1. Site coordinates, air pressure, and temperature of the sampling sites; Text S2. Chlorinated paraffin exposure measurement; Text S3. Covariates; Table S2. Distribution of sleep disorder and subtypes grouped by ∑CPs; Figure S2. Dose-response relationships of CPs concentrations in PM_2.5_ and sleep disorder risk; Table S3. Association between ∑CP mixture exposures and the risk of sleep disorder and subtypes among all participants in the WQS model; Table S4. Association between ∑CP mixture exposures and the risk of sleep disorder and subtypes among all participants in the qgcomp boot model; Figure S3. Estimated weights of individual CPs in the associations with the risk of sleep disorder and subtypes in the qgcomp models; Figure S4. GO and KEGG enrichment analysis of SCCP-related genes; Figure S5. Correlation analysis among sleep disorder score, BMI, and CP concentrations; Table S5. Association between ∑CP exposures and BMI among all participants in the logistic model; Table S6. Association between BMI and the risk of sleep disorder and subtypes among all participants in the logistic model; Table S7. Effects of CP exposure on sleep disorder and its subtypes via BMI; Table S8. Association between ∑CP exposures and the risk of sleep disorder and subtypes in the logistic model; Table S9. Association between ∑CP exposures and the risk of sleep disorder (t-score) and subtypes among all participants in the logistic model; Table S10 Association between standardized ∑CP exposures and the risk of sleep disorder and subtypes among all participants in the logistic model; Table S11. Association between ∑CP quantile levels and the risk of sleep disorder and subtypes among all participants in the logistic model; Table S12. Association between ∑CP quantile levels and the risk of sleep disorder and subtypes (t-score) among all participants in the logistic model; Table S13. Association between ∑CP exposures and the risk of sleep disorder and subtypes among participants without premature birth in the logistic model; Table S14. Association between ∑CP exposures and the risk of sleep disorder and subtypes among participants without breastfeeding in the logistic model; Table S15. Association between ∑CP exposures and the risk of sleep disorder and subtypes among participants without low birth weight in the logistic model.

## Abbreviations

The following abbreviations are used in this manuscript:

CPs	chlorinated paraffins
SCCPs	short-chain chlorinated paraffins
MCCPs	medium-chain chlorinated paraffins
LCCPs	long-chain chlorinated paraffins
PM_2.5_	fine particulate matter
PM_2.5_-bound CPs	fine particulate matter-bound chlorinated paraffins
SDSC	Chinese Sleep Disturbance Scale for Children
DIMS	sleep initiation and maintenance disorders
SWTD	sleep–wake transition disorders
DOES	excessive daytime sleepiness disorders
SBD	sleep breathing disorders
DA	disorders of arousal
SHY	sleep hyperhidrosis
GO	Gene Ontology
KEGG	Kyoto Encyclopedia of Genes and Genomes
GLMM	generalized linear mixed models
OR	odds ratio
95% CI	95% confidence interval
IQR	interquartile range
BMI	body mass index
PA	physical activity
SHS	secondhand smoke
RCS	Restricted cubic splines
qgcomp	Quantile g-computation
WQS	weighted quantile sum
