# Supplementary material for: Exposure to Emerging Contaminants Chlorinated Paraffins in PM2.5 and Sleep Disorders in Youth: Body Weight as a Mediator
Source: Toxics. 2026 Jul 11;14(7):607. doi: 10.3390/toxics14070607 (PMC13417338; doi:10.3390/toxics14070607)
Supplement: Supplementary file 1 [file toxics-14-00607-s001.zip › toxics-4401700-supplementary.pdf]

Supplementary information

Text S1 Study Recruitment Details 2
Figure S1 Schools and sampling site in the Pearl River Delta that were included in this study. 3
Table S1 Site coordinates, air pressure and temperature of the sampling site. 4
Text S2 Chlorinated Paraffin exposure measurement 6
Text S3 Covariates 7
Table S2 Distribution of sleep disorder and subtypes grouped by ∑CPs 8
Figure S2 Dose-response relationships of CPs concentrations in $PM_{2.5}$ and sleep disorder risk. 9
Table S3 Association between ∑CPs mixture exposures and the risk of sleep disorder and subtypes among all participants in the WQS model 10
Table S4 Association between ∑CPs mixture exposures and the risk of sleep disorder and subtypes among all participants in the qgcomp boot model. 11
Figure S3 Estimated weights of individual CPs in the associations with the risk of sleep disorder and subtypes with qgcomp models. 12
Figure S4 GO and KEGG enrichment analysis of SCCP-related genes. 13
Figure S5 Correlation analysis between sleep disorder score, BMI and CPs concentration. 14
Table S5 Association between ∑CPs exposures and BMI among all participants in the logistic model. 15
Table S6 Association between BMI and the risk of sleep disorder and subtypes among all participants in the logistic model. 16
Table S7 Effects CPs exposure on sleep disorder and its subtypes via BMI. 17
Table S8 Association between ∑CPs exposures and the risk of sleep disorder and subtypes in the logistic model **19**
Table S9 Association between ∑CPs exposures and the risk of sleep disorder (t-score) and subtypes among all participants in the logistic model 21
Table S10 Association between standardized ∑CP exposures and the risk of sleep disorder and subtypes among all participants i n the logistic model 24
Table S11 Association between ∑CPs quantile levels and the risk of sleep disorder and subtypes among all participants in the logistic model. 23
Table S12 Association between ∑CPs quantile levels and the risk of sleep disorder and subtypes (t-score) among all participants in the logistic model. 26
Table S13 Association between ∑CPs exposures and the risk of sleep disorder and subtypes among participants without premature birth in the logistic model. 27
Table S14 Association between ∑CPs exposures and the risk of sleep disorder and subtypes among participants without breastfeeding in the logistic model. 28
Table S15 Association between ∑CPs exposures and the risk of sleep disorder and subtypes among participants without low birth weight in the logistic model. 29

Text S1 Study Recruitment Details

A multi-stage random sampling method was employed in selecting the participants. The study covered six cities in PRD region of Guangdong Province, with 105 schools distributed as follows: 37 in Guangzhou, 11 in Shenzhen, 8 in Zhuhai, 18 in Foshan, 18 in Zhongshan, and 13 in Maoming. Figure S1 provides a map of the sampling locations, and Table S1 summarizes the specific information for each sampling site. Parents or guardians completed questionnaires related to social information, lifestyle, behaviors, environmental exposure, and medical history. Participants were excluded if they met any of the following criteria: (1) provided incomplete questionnaires; (2) had a history of congenital neurological disorders; or (3) reported a family history of such disorders.

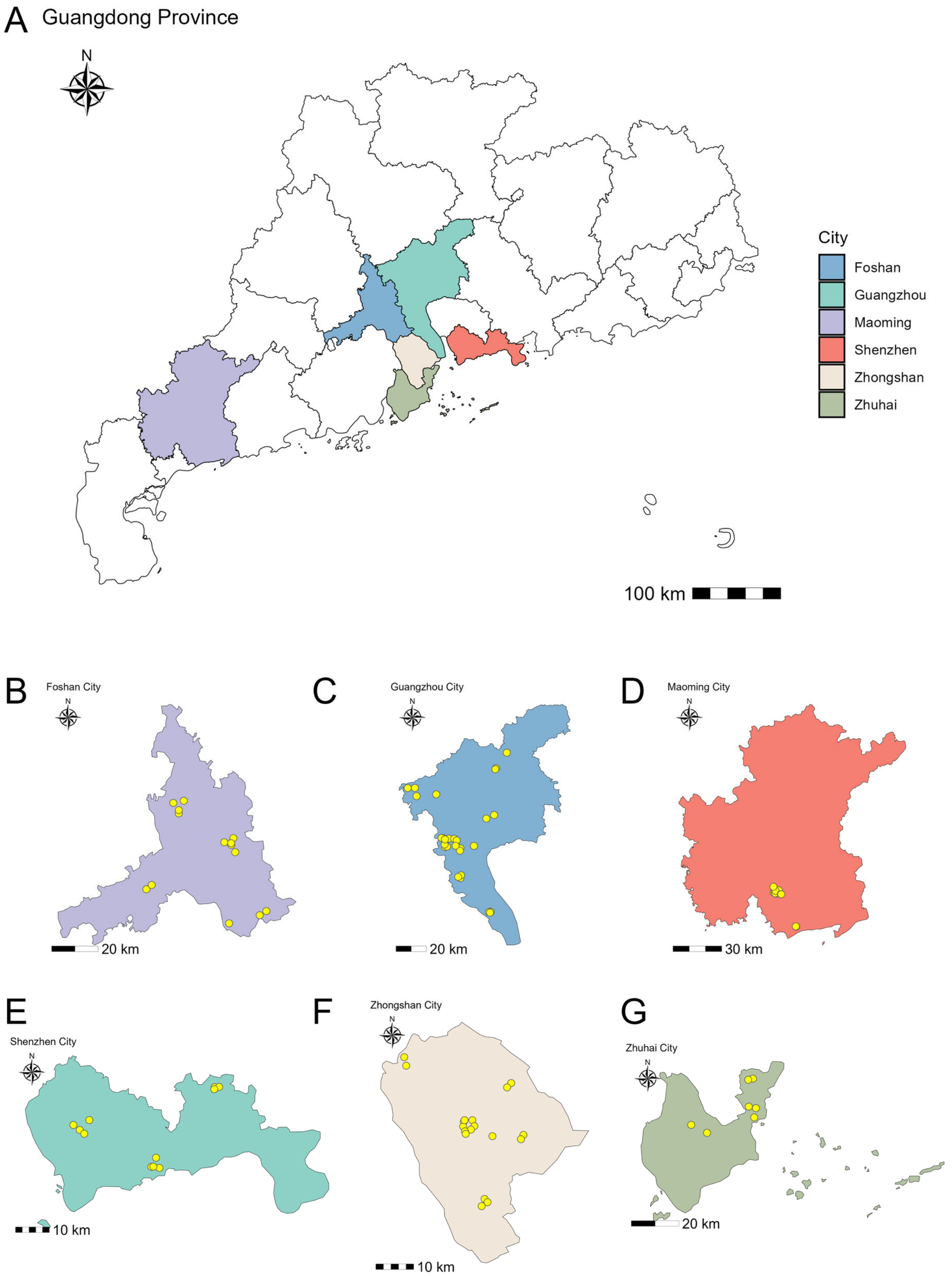

Figure S1 Schools and sampling site in the Pearl River Delta that were included in this study. Including Foshan (A), Guangzhou (B), Maoming, Shenzhen (C), Zhongshan (D), Zhuhai (E). Yellow circles represent the locations of sampling points collected in this study.

Table S1 Site coordinates, air pressure and temperature of the sampling site.

City	Site coordinates	Summer sampling		Winter sampling	
		Air pres. (kPa)	Temp. (Co)	Air pres. (kPa)	Temp. (Co)
Guangzhou	(113.5203, 23.2598)	100.7	32.9	101.5	26.4
Guangzhou	(113.2546, 23.1374)	101	33.7	101.9	29.3
Guangzhou	(113.2546, 23.1374)	101	33.7	101.9	29.3
Guangzhou	(113.5782, 23.5505)	100.8	27.5	100.8	34.8
Guangzhou	(113.3579, 22.9259)	101.9	27.7	101.99	22.1
Guangzhou	(113.2418, 23.1458)	101.1	30.3	101.7	27.9
Guangzhou	(113.6520, 23.6482)	100.5	30.8	101.9	29.9
Guangzhou	(113.3547, 23.0842)	101.7	30.8	101.8	27.6
Guangzhou	(113.3298, 23.1315)	100.8	24	101.9	31
Guangzhou	(113.1988, 23.4028)	101	35.6	102	29.2
Guangzhou	(113.2677, 23.0951)	101.6	30.6	101.6	25.9
Guangzhou	(113.5705, 23.2808)	100.9	30.7	102	27.5
Guangzhou	(113.3400, 22.9163)	101.3	27	101.8	24.9
Guangzhou	(113.2794, 23.1420)	101.1	30.6	101.5	24.9
Guangzhou	(113.3279, 23.1022)	101.7	34	101.4	26.3
Guangzhou	(113.5485, 22.7105)	100.5	29.6	102	27.3
Guangzhou	(113.0724, 23.3924)	101.2	31.2	101.9	28.7
Guangzhou	(113.4409, 23.0991)	101.2	29.3	101.2	29.5
Guangzhou	(113.3537, 22.9094)	101.5	27.1	101.8	24.9
Guangzhou	(113.5441, 22.7058)	100.5	29.2	102.1	26.3
Guangzhou	(113.0155, 23.4399)	100.3	33.4	101.9	31.6
Guangzhou	(113.2557, 23.0925)	101.3	28.1	102	34.3
Guangzhou	(113.3137, 23.1409)	101.1	32.1	101.8	27.6
Guangzhou	(113.0616, 23.4409)	100.8	36.5	98.5	32
Guangzhou	(113.3230, 23.0999)	100.8	26.9	101.9	32.7
Guangzhou	(113.3509, 23.0707)	101.4	29.7	100.8	22
Guangzhou	(113.5827, 23.5569)	100.5	27.5	101.9	29.9
Guangzhou	(113.2550, 23.1272)	101.2	30.7	101.3	26.9
Guangzhou	(113.2526, 23.1071)	101.1	28.7	100.8	23
Maoming	(110.8996, 21.6650)	101	26.3	101.5	29.8
Maoming	(110.9379, 21.6522)	100.6	30.2	101.2	31.5
Maoming	(111.0251, 21.4739)	101.1	27.1	101	29.1
Maoming	(110.9361, 21.6527)	101.4	27.2	101.4	29
Maoming	(110.8914, 21.6937)	100.9	30.6	101.4	32.6
Maoming	(111.0270, 21.4725)	101.1	30.6	101.3	28.3
Maoming	(110.8439, 21.9050)	101.5	27.1	101.3	27.3
Maoming	(110.9092, 21.6628)	101.3	26.7	101.5	29.6
Maoming	(110.8932, 21.6633)	101.2	25.4	101.6	33.6
Maoming	(110.9021, 21.6571)	101.5	27.1	101.4	28.7
Maoming	(110.9099, 21.6552)	101.2	26.8	101.3	31.2
Maoming	(110.9231, 21.6738)	101.2	29.6	101.2	32.6
Maoming	(110.9226, 21.6637)	101.3	28.1	101.3	30.2
Maoming	(110.9215, 21.6693)	101.4	27.1	101.5	30.4
Foshan	(112.7864, 22.8746)	100.4	24.2	102.6	23.2
Foshan	(112.7698, 22.8633)	100.4	24.2	102.3	27.7

Foshan	(113.1120, 22.7259)	101.1	28.7	99.4	26.6
Foshan	(112.9003, 23.1516)	101.2	27.7	97.1	22.4
Foshan	(113.1378, 23.0013)	101.3	26.4	102	26.5
Foshan	(113.2688, 22.7735)	101.2	27.9	102	27.1
Foshan	(113.1300, 23.0560)	101.1	27.6	101.5	25.1
Foshan	(113.1113, 23.0273)	101.2	27.9	101.5	27.7
Foshan	(113.1206, 23.0363)	101.5	27.8	101.8	27.3
Foshan	(113.1214, 23.0298)	100.8	28.6	101.4	26.4
Foshan	(112.9004, 23.1641)	101.4	28.5	101.8	22.5
Foshan	(113.2407, 22.7574)	101.4	29.9	102.4	26.1
Foshan	(113.0918, 23.0398)	101.2	28.8	101.4	25.6
Foshan	(112.9213, 23.2005)	101.6	25	101.7	22.2
Zhuhai	(113.5487, 22.2320)	100.9	34.3	100.3	29.6
Zhuhai	(113.5267, 22.2726)	101.3	28.5	101.3	31.1
Zhuhai	(113.5245, 22.3738)	100.8	32.2	102	22
Zhuhai	(113.2919, 22.2039)	101.3	29	101.9	21.6
Zhuhai	(113.5436, 22.3783)	100.8	33.2	101.8	24.7
Zhuhai	(113.5556, 22.2670)	100.2	29	101.6	28.7
Zhuhai	(113.3564, 22.1738)	109.5	39.8	101.3	27.7
Zhongshan	(113.3978, 22.5260)	100.7	33.7	101.7	24.5
Zhongshan	(113.3755, 22.5075)	100.6	32.5	102.4	25.2
Zhongshan	(113.3725, 22.5399)	100.5	35.3	100.8	37.5
Zhongshan	(113.3891, 22.5177)	100.7	31.3	100.8	21.9
Zhongshan	(113.4443, 22.5021)	100.5	35.1	101.9	23.1
Zhongshan	(113.3737, 22.5135)	100.6	32.3	102	26.6
Zhongshan	(113.4167, 22.3360)	100.5	34.4	102	25.2
Zhongshan	(113.3709, 22.5368)	100.6	34.4	102	26.8
Zhongshan	(113.3925, 22.5400)	100.7	33.6	102.1	27.6
Zhongshan	(113.3691, 22.5249)	100.8	33.8	100.8	36.6
Zhongshan	(113.4821, 22.6177)	100.8	35	102.3	28.9
Zhongshan	(113.4926, 22.6280)	100.7	32	102.5	26.7
Zhongshan	(113.2173, 22.6895)	100.7	35.1	102.4	29
Zhongshan	(113.5177, 22.4948)	100.9	32.5	101.8	25
Zhongshan	(113.4241, 22.3531)	101.2	34.7	101.9	27.3
Zhongshan	(113.4318, 22.3454)	100.6	36.2	102.5	23.5
Zhongshan	(113.2224, 22.6692)	100.6	35.8	102.4	28.6
Zhongshan	(113.5241, 22.5050)	100.7	33.3	102.5	23.6
Shenzhen	(113.8906, 22.6741)	100.4	36.5	101.6	29.1
Shenzhen	(113.9363, 22.6872)	100	34.2	100.9	30.5
Shenzhen	(114.3110, 22.7756)	100.5	36.7	100.8	28.3
Shenzhen	(114.1153, 22.5627)	100.9	36.1	101.1	28.2
Shenzhen	(114.1213, 22.5632)	100.7	33.8	102.3	25.1
Shenzhen	(114.1388, 22.5598)	101.1	35.2	101.8	27.4
Shenzhen	(114.1288, 22.5870)	100.7	34.7	101.2	26.2
Shenzhen	(114.2979, 22.7750)	100.9	35.6	101.4	31
Shenzhen	(113.9089, 22.6608)	100.7	36.7	101.3	29.7
Shenzhen	(114.2970, 22.7695)	100.9	35.4	97.9	24.5
Shenzhen	(113.9217, 22.6507)	100.8	25	100.8	26.9

Text S2 Chlorinated Paraffin exposure measurement

$PM_{2.5}$ samples were collected during summer (May-July) and winter (December) of 2018 using medium-volume (TH-150 C/D; Tianhong Instrument Co., Ltd. Wuhan, China) and high-volume (TH-1000 H; Tianhong Instrument Co., Ltd. Wuhan, China) air samplers with quartz fiber filters (Whatman Inc.). A portion (1/4 or 1/8) of each filter was spiked with 5 ng of 13C10-trans-chlordane (Ehrenstorfer GmbH, Augsburg, Germany) as internal standard, followed by ultrasonic extraction and purification via multi-layer Florisil silica gel (63–100 µm; Merck, Whitehouse Station, NJ, USA) chromatography. Quantification of SCCPs, MCCPs, and LCCPs (Ehrenstorfer GmbH, Augsburg, Germany) was performed using UPLC-QTOF-MS (X500R, AB Sciex, Canada) with an ESI source. Quality assurance included blank correction (sample concentrations subtracted by average blank values) and substitution of concentrations below LOD with LOD/√2. The limits of quantification (LOQ), defined as ten times the standard deviation of blank concentrations, were 20.5 ng for SCCPs, 16.5 ng for MCCPs, and 5.6 ng for LCCPs.

Text S3 Covariates

The main GLMM model adjusted for variables such as sex, age, body mass index (BMI) category, maternal age, cesarean delivery, preterm birth, low birth weight, breastfeeding status, parental education, household income, health insurance, physical activity (PA), secondhand smoke (SHS) exposure, pet ownership, and $PM_{2.5}$ levels. Additionally, city was included as a random effect to address for potential regional clustering. RCS model, mediation analyses and mixture analysis were adjusted the same covariates with the main model.

Table S2 Distruibution of sleep disorder and subtypes grouped by ∑CPs

Variables	Low ∑CP n= 61407	High ∑CP n=61558	*P* value
Sleep disorder, n (%)	2704 (4.4)	3205 (5.2)	<0.001
DIMS, n (%)	3696 (6.0)	4672 (7.6)	<0.001
SBD, n (%)	2440 (4.0)	3215 (5.2)	<0.001
DA, n (%)	1630 (2.7)	2660 (4.3)	<0.001
SWTD, n (%)	2220 (3.6)	2761 (4.5)	<0.001
DOSE, n (%)	2657 (4.3)	3581 (5.8)	<0.001
SHY, n (%)	3323 (5.4)	4820 (7.8)	<0.001
Short sleep duration, n (%)	4561 (7.4)	16869 (27.4)	<0.001
Long sleep platency, n (%)	934 (1.5)	3503 (5.7)	<0.001
Total t-score, mean (SD)	50.88 (10.19)	52.79 (9.96)	<0.001
DIMS t-score, mean (SD)	52.48 (10.73)	54.15 (10.57)	<0.001
SBD t-score, mean (SD)	48.75 (9.56)	51.16 (9.67)	<0.001
DA t-score, mean (SD)	49.17 (9.18)	51.26 (8.97)	<0.001
SWTD t-score, mean (SD)	49.20 (9.90)	51.38 (9.74)	<0.001
DOES t-score, mean (SD)	50.49 (9.91)	52.22 (9.54)	<0.001
SHY t-score, mean (SD)	50.19 (10.27)	53.45 (10.46)	<0.001

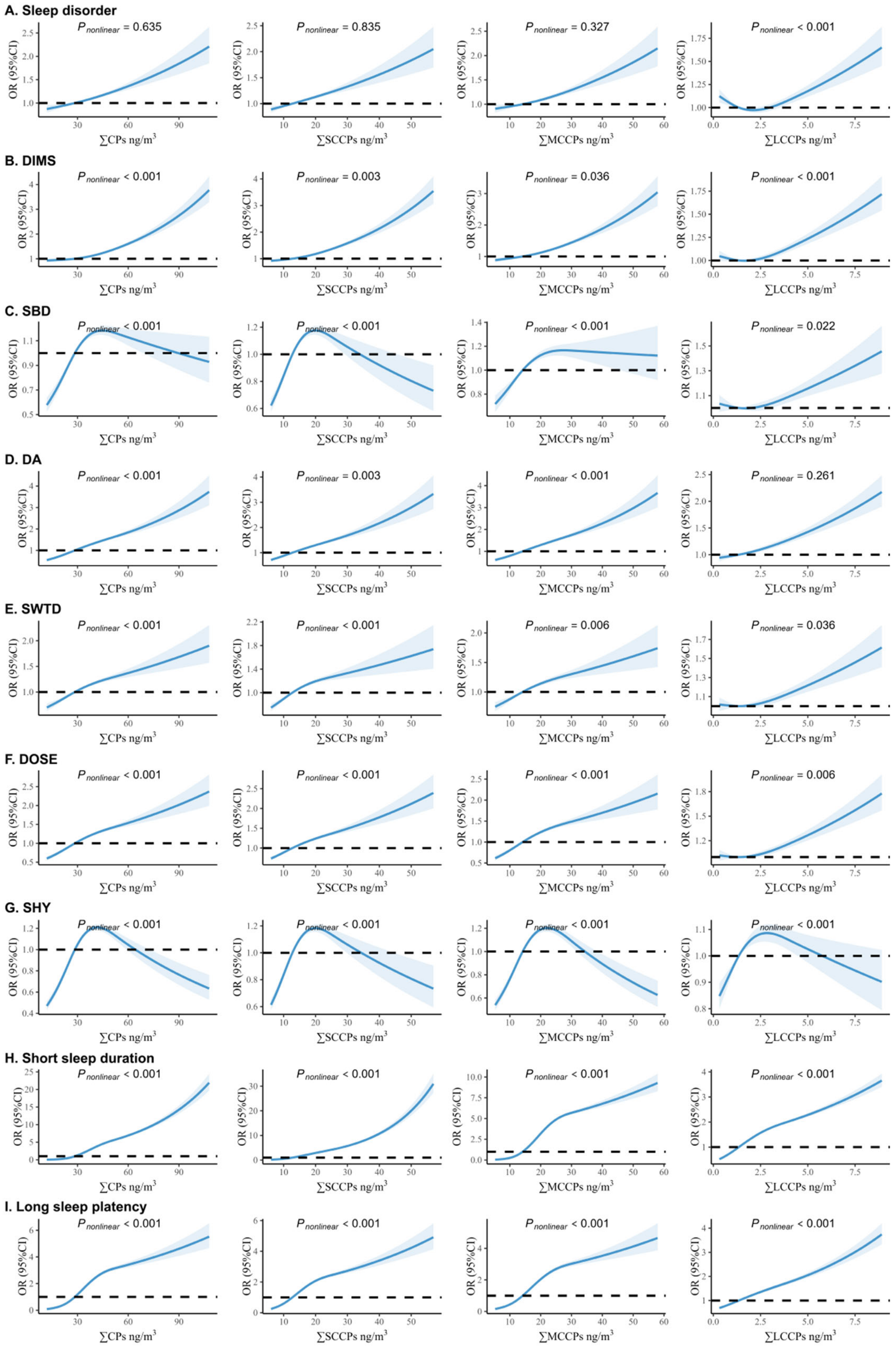

Figure S2 Dose-response relationships of CPs concentrations in $PM_{2.5}$ and sleep disorder risk. ORs were calculated by RCS model after adjusting for sex, age, BMI class, physical activity, low birth weight, premature birth, caesarean, breastfeeding, parental education, maternal age, insurance, family income class, second hand smoking exposure, petting ownership and .

Table S3 Association between ∑CPs mixture exposures and the risk of sleep disorder and subtypes among all participants in the WQS model.

Variables	OR (95% CI)	*P* value
Categorical variables		
Sleep disorder	1.13 (1.09, 1.17)	<0.001
DIMS	1.18 (1.15, 1.22)	<0.001
SBD	1.42 (1.36, 1.48)	<0.001
DA	1.17 (1.13, 1.22)	<0.001
SWTD	1.21 (1.16, 1.26)	<0.001
DOSE	1.27 (1.23, 1.31)	<0.001
SHY	1.21 (1.17, 1.24)	<0.001
Short sleep duration	3.61 (3.51, 3.72)	<0.001
Long sleep platency	2.41 (2.29, 2.53)	<0.001

WQS Model: Adjusted for sex, age, BMI class, physical activity, low birth weight, premature birth, caesarean, breastfeeding, parental education, maternal age, insurance, family income class, second hand smoking exposure, petting ownership and $PM_{2.5}$ concentration

Table S4 Association between ∑CPs mixture exposures and the risk of sleep disorder and subtypes among all participants in the qgcomp boot model.

Categorical variables	RR (95% CI)	*P* value
Sleep disorder	1.12 (1.08, 1.15)	<0.001
DIMS	1.18 (1.15, 1.21)	<0.001
SBD	1.16 (1.13, 1.20)	<0.001
DA	1.38 (1.32, 1.44)	<0.001
SWTD	1.19 (1.15, 1.24)	<0.001
DOSE	1.25 (1.21, 1.29)	<0.001
SHY	1.21 (1.18, 1.24)	<0.001
Short sleep duration	2.98 (2.93, 3.04)	<0.001
Long sleep platency	2.48 (2.38, 2.59)	<0.001

Model: Adjusted for sex, age, BMI class, physical activity, low birth weight, premature birth, caesarean, breastfeeding, parental education, maternal age, insurance, family income class, second hand smoking exposure, petting ownership and $PM_{2.5}$ concentration.

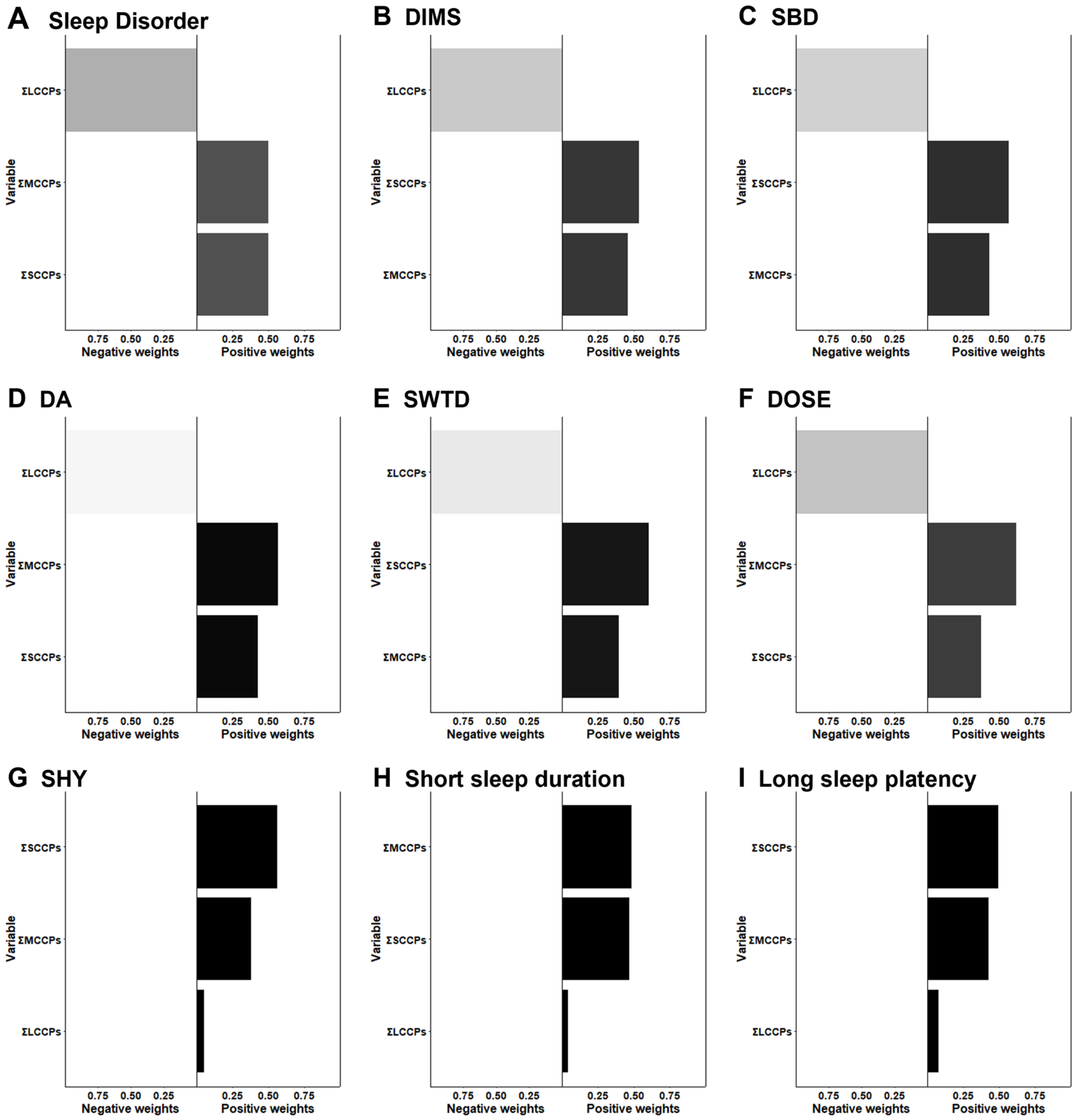

Figure S3 Estimated weights of individual CPs in the associations with the risk of sleep disorder and subtypes with qgcomp models.

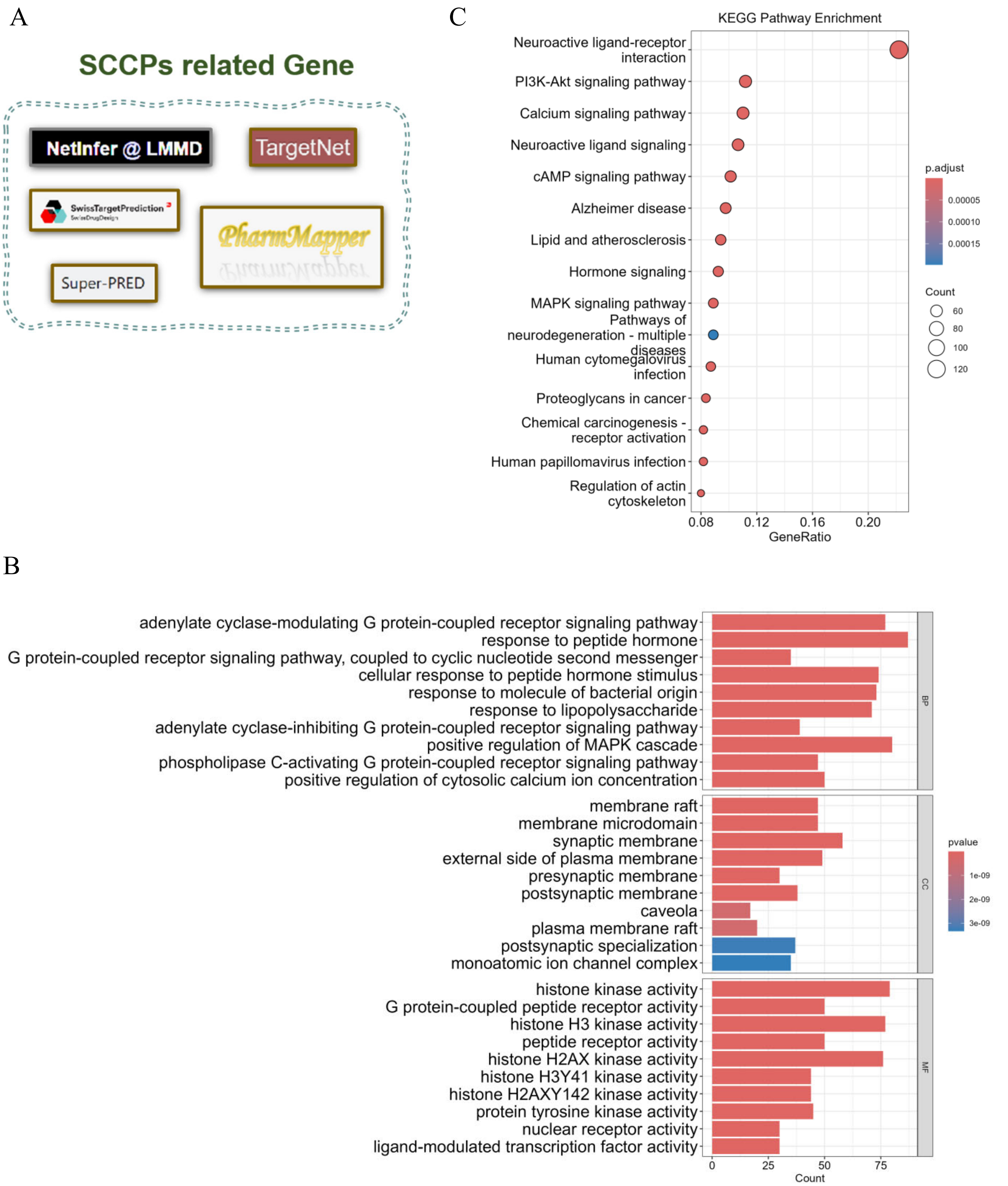

Figure S4 GO and KEGG enrichment analysis of SCCP-related genes.

(A) Flowchart of SCCP-related gene selection and analysis

(B) GO enrichment analysis showing biological processes, molecular functions, and cellular components.

(C) KEGG pathway enrichment analysis.

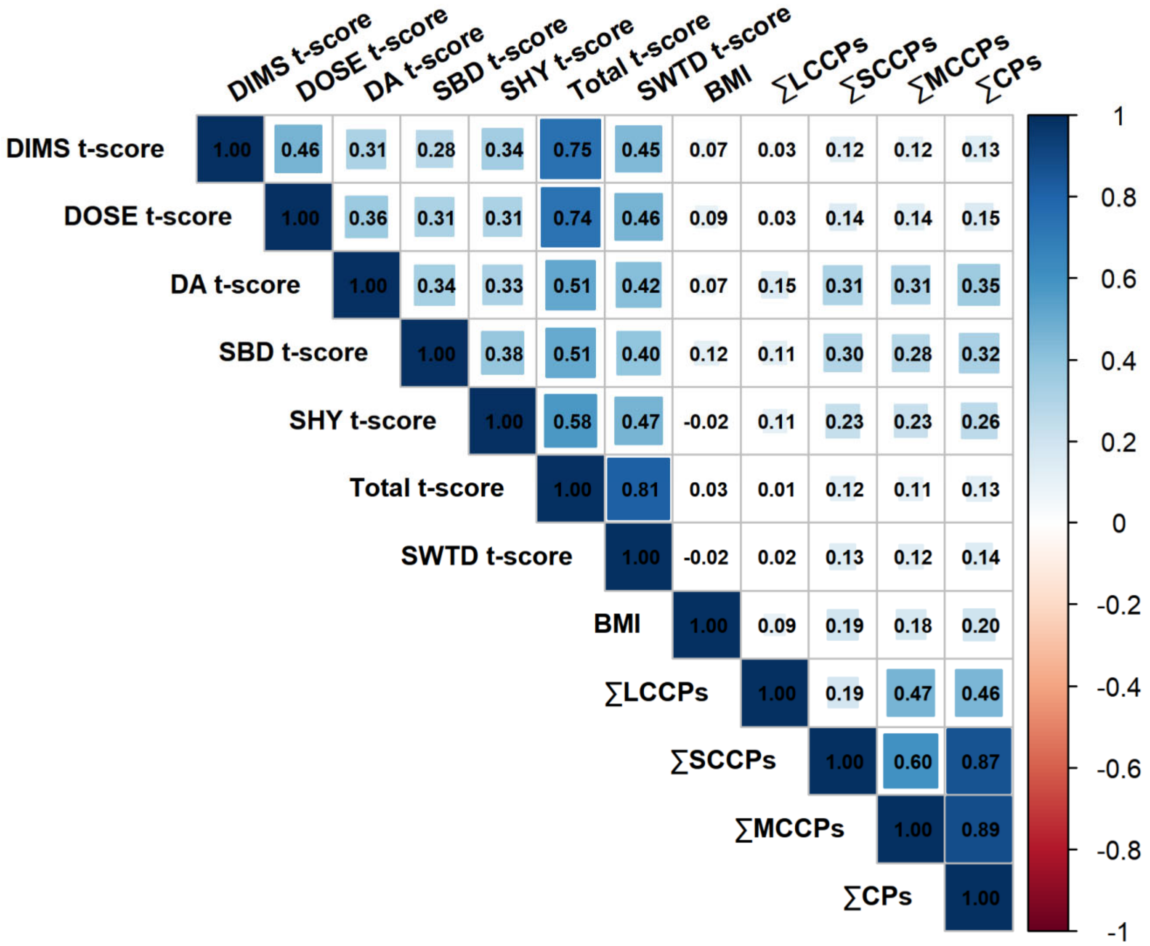

Figure S5 Correlation analysis between sleep disorder score, BMI and CPs concentration.

Table S5 Association between ∑CPs exposures and BMI among all participants in the logistic model.

Variables	*β (95% CI*)	*P* value
∑CPs	0.70 (0.68, 0.72)	<0.001
∑SCCPs	0.62 (0.60, 0.64)	<0.001
∑MCCPs	0.34 (0.32, 0.35)	<0.001
∑LCCPs	0.81 (0.79, 0.83)	<0.001

Model: Adjusted for sex, age, physical activity, low birth weight, premature birth, caesarean, breastfeeding, parental education, maternal age, insurance, family income class, second hand smoking exposure, petting ownership and $PM_{2.5}$ concentration.

Table S6 Association between BMI and the risk of sleep disorder and subtypes among all participants in the logistic model.

Variables	*OR or β (95% CI)*	*P* value
Categorical variables (*OR, 95% CI*)		
Sleep disorder	1.04 (1.03, 1.06)	<0.001
DIMS	1.03 (1.02, 1.04)	<0.001
SBD	1.06 (1.05, 1.07)	<0.001
DA	1.04 (1.03, 1.06)	<0.001
SWTD	1.03 (1.02, 1.05)	<0.001
DOSE	1.04 (1.03, 1.06)	<0.001
SHY	1.02 (1.01, 1.03)	<0.001
Short sleep duration	1.16 (1.15, 1.17)	<0.001
Long sleep platency	1.10 (1.08, 1.11)	<0.001
Continuous variables (*β, 95% CI*)		
Total t-score	0.15 (0.13, 0.16)	<0.001
DIMS t-score	0.13 (0.11, 0.15)	<0.001
SBD t-score	0.31 (0.29, 0.32)	<0.001
DA t-score	0.11 (0.09, 0.13)	<0.001
SWTD t-score	0.10 (0.08, 0.11)	<0.001
DOES t-score	0.15 (0.13, 0.17)	<0.001
SHY t-score	0.15 (0.13, 0.17)	<0.001

Model: Adjusted for sex, age, physical activity, low birth weight, premature birth, caesarean, breastfeeding, parental education, maternal age, insurance, family income class, second hand smoking exposure and petting ownership.

Table S7 Effects CPs exposure on sleep disorder and its subtypes via BMI.

Pollutants	outcome	Indirect effect (95% CI)	Direct effect (95% CI)	Proportions (%)
∑CPs	Sleep disorder	**1.0009 (1.0006-1.0011)**	**1.0051 (1.0043-1.0059)**	**14.41**
	DIMS	1.0003 (1.0000-1.0005)	**1.0104 (1.0097-1.0110)**	2.59
	SBD	**1.0018 (1.0015-1.0020)**	**1.0032 (1.0023-1.0039)**	**35.67**
	DA	**1.0002 (1.0001-1.0004)**	**1.0065 (1.0062-1.0068)**	**3.40**
	SWTD	**1.0003 (1.0001-1.0006)**	**1.0051 (1.0044-1.0056)**	**6.46**
	DOSE	**1.0006 (1.0004-1.0008)**	**1.0079 (1.0073-1.0084)**	**7.05**
	SHY	**1.0006 (1.0002-1.0009)**	**1.0053 (1.0041-1.0062)**	**9.66**
	Short sleep duration	**1.0016 (1.0014-1.0017)**	**1.0364 (1.0358-1.0370)**	**4.18**
	Long sleep platency	**1.0003 (1.0001-1.0004)**	**1.0073 (1.0071-1.0075)**	**3.42**
∑SCCPs				
	Sleep disorder	**1.0008 (1.0006-1.0011)**	**1.0046 (1.0037-1.0054)**	**15.32**
	DIMS	**1.0003 (1.0001-1.0006)**	**1.0099 (1.0091-1.0106)**	**3.36**
	SBD	**1.0016 (1.0014-1.0019)**	**1.0029 (1.0021-1.0037)**	**35.92**
	DA	**1.0004 (1.0002-1.0005)**	**1.0059 (1.0055-1.0063)**	**5.61**
	SWTD	**1.0004 (1.0002-1.0006)**	**1.0048 (1.0040-1.0054)**	**7.23**
	DOSE	**1.0007 (1.0004-1.0009)**	**1.0071 (1.0065-1.0077)**	**8.49**
	SHY	**1.0005 (1.0003-1.0008)**	**1.0058 (1.0047-1.0067)**	**8.01**
	Short sleep duration	**1.0023 (1.0021-1.0026)**	**1.0456 (1.0450-1.0463)**	**5.00**
	Long sleep platency	**1.0004 (1.0003-1.0005)**	**1.0078 (1.0075-1.0081)**	**5.13**
∑MCCPs				
	Sleep disorder	**1.0007 (1.0005-1.0010)**	**1.0042 (1.0035-1.0049)**	**14.90**
	DIMS	**1.0004 (1.0001-1.0006)**	**1.0085 (1.0077-1.0092)**	**4.38**
	SBD	**1.0014 (1.0012-1.0016)**	**1.0025 (1.0016-1.0032)**	**36.22**
	DA	**1.0003 (1.0001-1.0004)**	**1.0058 (1.0054-1.0060)**	**4.26**
	SWTD	**1.0004 (1.0002-1.0005)**	**1.0040 (1.0034-1.0046)**	**8.13**
	DOSE	**1.0006 (1.0004-1.0008)**	**1.0069 (1.0063-1.0075)**	**7.69**
	SHY	**1.0005 (1.0003-1.0008)**	**1.0036 (1.0026-1.0045)**	**13.11**
	Short sleep duration	**1.0023 (1.0021-1.0025)**	**1.0380 (1.0375-1.0385)**	**5.81**
	Long sleep platency	**1.0003 (1.0003-1.0004)**	**1.0069 (1.0067-1.0071)**	**4.76**
∑LCCPs				
	Sleep disorder	**1.0006 (1.0005-1.0007)**	**1.0020 (1.0012-1.0028)**	**22.06**
	DIMS	**1.0004 (1.0003-1.0006)**	**1.0040 (1.0031-1.0050)**	**10.00**
	SBD	**1.0009 (1.0008-1.0011)**	**1.0016 (1.0008-1.0023)**	**37.12**
	DA	**1.0003 (1.0002-1.0004)**	**1.0035 (1.0030-1.0040)**	**8.54**
	SWTD	**1.0003 (1.0002-1.0004)**	**1.0023 (1.0014-1.0029)**	**11.85**

DOSE	**1.0006 (1.0004-1.0007)**	**1.0033 (1.0025-1.0041)**	**14.65**
SHY	**1.0004 (1.0003-1.0006)**	1.0007 (0.9997-1.0018)	**37.41**
Short sleep duration	**1.0042 (1.0040-1.0044)**	**1.0290 (1.0280-1.0299)**	**12.72**
Long sleep platency	**1.0005 (1.0005-1.0006)**	**1.0062 (1.0058-1.0066)**	**8.14**

Model: Medition analysis models were adjusted for sex, age, physical activity, low birth weight, premature birth, caesarean, breastfeeding, parental education, maternal age, insurance, family income class, second hand smoking exposure and petting ownership and $PM_{2.5}$ concentration.

Table S8 Association between ∑CPs exposures and the risk of sleep disorder and subtypes in the logistic model

Variables	Boys	Girls	*P* *interaction*	Age ≤12	Age > 12	*P* *interaction*
∑CPs						
Sleep disorder	**1.11 (1.06, 1.17)**	**1.16 (1.13, 1.19)**	**<0.001**	**1.20 (1.16, 1.24)**	**1.12 (1.09, 1.16)**	**<0.001**
DIMS	**1.19 (1.14, 1.24)**	**1.27 (1.24, 1.30)**	**<0.001**	**1.35 (1.31, 1.39)**	**1.22 (1.19, 1.25)**	**<0.001**
SBD	**1.04 (0.99, 1.09)**	**1.07 (1.04, 1.10)**	0.124	**1.10 (1.06, 1.13)**	**1.04 (1.01, 1.07)**	**0.006**
DA	**1.08 (1.02, 1.13)**	**1.23 (1.20, 1.27)**	**0.002**	**1.31 (1.27, 1.36)**	**1.23 (1.19, 1.27)**	**0.001**
SWTD	**1.09 (1.03, 1.14)**	**1.15 (1.12, 1.19)**	**0.001**	**1.19 (1.15, 1.23)**	**1.14 (1.10, 1.17)**	**0.028**
DOSE	**1.11 (1.06, 1.17)**	**1.21 (1.18, 1.24)**	**<0.001**	**1.23 (1.19, 1.28)**	**1.20 (1.17, 1.23)**	0.091
SHY	**1.06 (1.01, 1.10)**	**1.12 (1.09, 1.15)**	**<0.001**	**1.15 (1.11, 1.18)**	**1.09 (1.06, 1.13)**	**0.005**
Short sleep duration	**1.74 (1.69, 1.8)**	**2.62 (2.57, 2.68)**	**<0.001**	**2.47 (2.41, 2.53)**	**3.06 (2.99, 3.13)**	**<0.001**
Long sleep platency	**1.17 (1.11, 1.22)**	**1.51 (1.47, 1.55)**	**<0.001**	**1.70 (1.64, 1.76)**	**1.55 (1.51, 1.59)**	**<0.001**
∑SCCPs						
Sleep disorder	**1.19 (1.11, 1.28)**	**1.18 (1.14, 1.22)**	0.618	**1.22 (1.17, 1.26)**	**1.13 (1.10, 1.16)**	**<0.001**
DIMS	**1.36 (1.28, 1.44)**	**1.31 (1.28, 1.35)**	0.073	**1.38 (1.34, 1.42)**	**1.23 (1.20, 1.26)**	**<0.001**
SBD	**1.1 (1.02, 1.18)**	**1.08 (1.05, 1.12)**	0.587	**1.11 (1.08, 1.15)**	**1.04 (1.01, 1.07)**	**<0.001**
DA	**1.25 (1.16, 1.36)**	**1.26 (1.21, 1.31)**	0.840	**1.35 (1.30, 1.41)**	**1.23 (1.19, 1.26)**	**<0.001**
SWTD	**1.18 (1.09, 1.29)**	**1.17 (1.13, 1.22)**	0.739	**1.21 (1.16, 1.25)**	**1.14 (1.10, 1.17)**	**0.002**
DOSE	**1.21 (1.13, 1.30)**	**1.23 (1.19, 1.27)**	0.606	**1.26 (1.21, 1.31)**	**1.20 (1.17, 1.23)**	**0.005**
SHY	**1.13 (1.06, 1.21)**	**1.13 (1.10, 1.17)**	0.997	**1.16 (1.13, 1.19)**	**1.09 (1.05, 1.12)**	**<0.001**
Short sleep duration	**2.59 (2.45, 2.73)**	**2.74 (2.67, 2.81)**	**0.001**	**2.64 (2.57, 2.70)**	**2.83 (2.78, 2.89)**	**<0.001**
Long sleep platency	**1.68 (1.57, 1.80)**	**1.63 (1.57, 1.68)**	0.163	**1.84 (1.78, 1.91)**	**1.54 (1.50, 1.58)**	**<0.001**
∑MCCPs						
Sleep disorder	**1.14 (1.10, 1.17)**	**1.17 (1.14, 1.20)**	**0.002**	**1.18 (1.15, 1.22)**	**1.12 (1.08, 1.15)**	**0.001**
DIMS	**1.23 (1.20 , 1.26)**	**1.29 (1.27, 1.32)**	**<0.001**	**1.32 (1.29, 1.35)**	**1.22 (1.19, 1.25)**	**<0.001**
SBD	**1.06 (1.02, 1.09)**	**1.08 (1.05, 1.11)**	**0.040**	**1.08 (1.05, 1.12)**	**1.04 (1.01, 1.08)**	**0.028**

DA	**1.17 (1.13, 1.21)**	**1.28 (1.25, 1.32)**	**<0.001**	**1.29 (1.24, 1.33)**	**1.23 (1.19, 1.27)**	**0.022**
SWTD	**1.13 (1.09, 1.17)**	**1.17 (1.14, 1.21)**	**<0.001**	**1.17 (1.14, 1.21)**	**1.14 (1.10, 1.18)**	0.104
DOSE	**1.17 (1.14, 1.21)**	**1.24 (1.21, 1.27)**	**<0.001**	**1.22 (1.19, 1.26)**	**1.20 (1.17, 1.24)**	0.317
SHY	**1.08 (1.05, 1.12)**	**1.14 (1.11, 1.17)**	**<0.001**	**1.13 (1.10, 1.16)**	**1.10 (1.07, 1.14)**	0.105
Short sleep duration	**2.28 (2.23, 2.33)**	**2.93 (2.87, 2.99)**	**<0.001**	**2.52 (2.47, 2.57)**	**3.19 (3.11, 3.26)**	**<0.001**
Long sleep platency	**1.36 (1.32, 1.4)**	**1.65 (1.61, 1.69)**	**<0.001**	**1.61 (1.57, 1.66)**	**1.58 (1.54, 1.63)**	0.265
∑LCCPs						
Sleep disorder	**1.16 (1.13, 1.19)**	**1.18 (1.15, 1.21)**	0.070	**1.15 (1.12, 1.19)**	**1.16 (1.12, 1.20)**	0.827
DIMS	**1.27 (1.24, 1.30)**	**1.31 (1.28, 1.34)**	**<0.001**	**1.27 (1.25, 1.30)**	**1.26 (1.23, 1.30)**	0.509
SBD	**1.07 (1.04, 1.10)**	**1.08 (1.05, 1.11)**	0.418	**1.06 (1.03, 1.09)**	**1.09 (1.05, 1.13)**	0.108
DA	**1.24 (1.20, 1.27)**	**1.29 (1.25, 1.33)**	**<0.001**	**1.25 (1.22, 1.29)**	**1.29 (1.25, 1.34)**	**0.040**
SWTD	**1.15 (1.12, 1.19)**	**1.18 (1.15, 1.22)**	**0.002**	**1.15 (1.12, 1.18)**	**1.19 (1.15, 1.23)**	**0.034**
DOSE	**1.22 (1.19, 1.25)**	**1.25 (1.22, 1.28)**	**<0.001**	**1.20 (1.17, 1.23)**	**1.23 (1.20, 1.27)**	0.050
SHY	**1.14 (1.11, 1.16)**	**1.13 (1.10, 1.16)**	0.378	**1.12 (1.10, 1.15)**	**1.12 (1.08, 1.16)**	0.760
Short sleep duration	**2.7 (2.64, 2.75)**	**2.97 (2.91, 3.03)**	**<0.001**	**2.74 (2.69, 2.80)**	**3.30 (3.22, 3.38)**	<0.001
Long sleep platency	**1.54 (1.51, 1.58)**	**1.70 (1.66, 1.74)**	**<0.001**	**1.56 (1.52, 1.60)**	**1.70 (1.65, 1.76)**	<0.001

Model 1: Adjusted for BMI class, physical activity, low birth weight, premature birth, caesarean, breastfeeding, parental education, maternal age, insurance, family income class, second hand smoking exposure and petting ownership

Bold: *P < 0.05*

Table S9 Association between ∑CPs exposures and the risk of sleep disorder (t-score) and subtypes among all participants in the logistic model.

Variables	Boys	Girls	*P interaction*	Age ≤12	Age > 12	*P interaction*
∑CPs						
Total t-score	**0.69 (0.58, 0.80)**	**1.06 (1, 10.13)**	**<0.001**	**1.29 (1.22, 1.36)**	**1.03 (0.96, 1.10)**	**<0.001**
DIMS t-score	**0.73 (0.61, 0.85)**	**1.28 (1.21, 1.35)**	**<0.001**	**1.54 (1.46, 1.62)**	**1.30 (1.22, 1.37)**	**<0.001**
SBD t-score	**0.55 (0.44, 0.66)**	**0.97 (0.91, 1.03)**	**<0.001**	**1.18 (1.11, 1.25)**	**0.98 (0.91, 1.04)**	**<0.001**
DA t-score	**0.51 (0.41, 0.61)**	**1.02 (0.96, 1.08)**	**<0.001**	**1.25 (1.18, 1.32)**	**1.04 (0.98, 1.11)**	**<0.001**
SWTD t-score	**0.64 (0.53, 0.75)**	**1.03 (0.97, 1.10)**	**<0.001**	**1.25 (1.18, 1.32)**	**1.03 (0.96, 1.09)**	**<0.001**
DOES t-score	**0.67 (0.56, 0.78)**	**1.02 (0.96, 1.08)**	**<0.001**	**1.11 (1.04, 1.18)**	**1.10 (1.03, 1.17)**	0.845
SHY t-score	**0.86 (0.75, 0.97)**	**1.50 (1.44, 1.57)**	**<0.001**	**1.78 (1.71, 1.85)**	**1.55 (1.48, 1.62)**	**<0.001**
∑SCCPs						
Total t-score	**1.34 (1.16, 1.52)**	**1.21 (1.13, 1.30)**	**0.029**	**1.41 (1.33, 1.49)**	**1.01 (0.94, 1.08)**	**<0.001**
DIMS t-score	**1.60 (1.41, 1.79)**	**1.47 (1.39, 1.56)**	**0.032**	**1.71 (1.62, 1.80)**	**1.25 (1.18, 1.32)**	**<0.001**
SBD t-score	**1.18 (1.01, 1.35)**	**1.11 (1.03, 1.19)**	0.195	**1.32 (1.24, 1.40)**	**0.94 (0.87, 1.00)**	**<0.001**
DA t-score	**1.16 (1.00, 1.32)**	**1.15 (1.07, 1.22)**	0.823	**1.39 (1.32, 1.46)**	**1.00 (0.94, 1.07)**	**<0.001**
SWTD t-score	**1.24 (1.07, 1.41)**	**1.17 (1.09, 1.25)**	0.187	**1.36 (1.28, 1.44)**	**1.00 (0.94, 1.07)**	**<0.001**
DOES t-score	**1.27 (1.10, 1.44)**	**1.16 (1.08, 1.24)**	**0.049**	**1.18 (1.10, 1.26)**	**1.06 (1.00, 1.13)**	**0.004**
SHY t-score	**1.75 (1.57, 1.93)**	**1.69 (1.61, 1.77)**	0.261	**1.95 (1.87, 2.03)**	**1.5 (1.43, 1.57)**	**<0.001**
∑MCCPs						
Total t-score	**0.90 (0.83, 0.97)**	**1.17 (1.11, 1.23)**	**<0.001**	**1.21 (1.14, 1.28)**	**1.07 (0.99, 1.14)**	**<0.001**
DIMS t-score	**1.05 (0.97, 1.13)**	**1.43 (1.37, 1.50)**	**<0.001**	**1.44 (1.37, 1.51)**	**1.36 (1.28, 1.44)**	**0.043**
SBD t-score	**0.81 (0.74, 0.88)**	**1.09 (1.03, 1.15)**	**<0.001**	**1.11 (1.05, 1.17)**	**1.02 (0.95, 1.09)**	**0.017**
DA t-score	**0.82 (0.75, 0.89)**	**1.16 (1.11, 1.22)**	**<0.001**	**1.17 (1.11, 1.23)**	**1.10 (1.03, 1.17)**	**0.031**
SWTD t-score	**0.87 (0.80, 0.94)**	**1.15 (1.09, 1.21)**	**<0.001**	**1.18 (1.11, 1.25)**	**1.06 (0.98, 1.13)**	**0.001**
DOES t-score	**0.87 (0.80, 0.94)**	**1.12 (1.06, 1.18)**	**<0.001**	**1.08 (1.01, 1.15)**	**1.14 (1.07, 1.21)**	0.117
SHY t-score	**1.23 (1.15, 1.31)**	**1.69 (1.62, 1.75)**	**<0.001**	**1.68 (1.61, 1.75)**	**1.62 (1.54, 1.69)**	0.078
∑LCCPs						
Total t-score	**1.09 (1.03, 1.15)**	**1.20 (1.14, 1.27)**	**<0.001**	**1.14 (1.08, 1.20)**	**1.20 (1.12, 1.27)**	0.066

DIMS t-score	**1.31 (1.24, 1.38)**	**1.50 (1.43, 1.57)**	**<0.001**	**1.39 (1.32, 1.46)**	**1.51 (1.42, 1.59)**	**<0.001**
SBD t-score	**0.99 (0.93, 1.05)**	**1.15 (1.09, 1.21)**	**<0.001**	**1.05 (0.99, 1.11)**	**1.2 (1.13, 1.27)**	**<0.001**
DA t-score	**1.06 (1.00, 1.12)**	**1.21 (1.16, 1.27)**	**<0.001**	**1.12 (1.06, 1.18)**	**1.23 (1.16, 1.30)**	**<0.001**
SWTD t-score	**1.07 (1.01, 1.13)**	**1.18 (1.12, 1.24)**	**<0.001**	**1.11 (1.05, 1.17)**	**1.2 (1.13, 1.28)**	**0.002**
DOES t-score	**1.04 (0.98, 1.10)**	**1.16 (1.10, 1.22)**	**<0.001**	**1.09 (1.03, 1.15)**	**1.19 (1.11, 1.26)**	**0.001**
SHY t-score	**1.57 (1.51, 1.63)**	**1.73 (1.67, 1.80)**	**<0.001**	**1.64 (1.58, 1.70)**	**1.75 (1.67, 1.83)**	**<0.001**

Model 1: Adjusted for BMI class, physical activity, low birth weight, premature birth, caesarean, breastfeeding, parental education, maternal age, insurance, family income class, second hand smoking exposure, petting ownership and $PM_{2.5}$ concentration.

Bold: $P < 0.05$

Table S10 Association between standardized ∑CPs exposures and the risk of sleep disorder and subtypes among all participants in the logistic model.

Variables	∑CPs SD	∑SCCPs SD	∑MCCPs SD	∑LCCPs SD
Categorical variables (OR, 95% CI)				
Sleep disorder	**1.18 (1.15, 1.21)**	**1.17 (1.14, 1.20)**	**1.16 (1.12, 1.19)**	**1.08 (1.06, 1.12)**
DIMS	**1.32 (1.29, 1.35)**	**1.28 (1.26, 1.31)**	**1.27 (1.24, 1.30)**	**1.15 (1.12, 1.18)**
SBD	**1.09 (1.06, 1.12)**	**1.07 (1.05, 1.10)**	**1.08 (1.05, 1.11)**	**1.04 (1.01, 1.07)**
DA	**1.33 (1.29, 1.37)**	**1.26 (1.22, 1.29)**	**1.30 (1.26, 1.34)**	**1.16 (1.13, 1.20)**
SWTD	**1.19 (1.16, 1.23)**	**1.17 (1.13, 1.20)**	**1.16 (1.13, 1.20)**	**1.11 (1.07, 1.14)**
DOSE	**1.27 (1.24, 1.30)**	**1.23 (1.20, 1.26)**	**1.24 (1.21, 1.27)**	**1.12 (1.09, 1.15)**
SHY	**1.15 (1.12, 1.17)**	**1.13 (1.10, 1.16)**	**1.14 (1.11, 1.16)**	**1.03 (1.01, 1.06)**
Short sleep duration	**3.08 (3.02, 3.15)**	**2.77 (2.72, 2.83)**	**2.43 (2.39, 2.48)**	**1.50 (1.48, 1.53)**
Long sleep platency	**1.80 (1.75, 1.84)**	**1.59 (1.55, 1.63)**	**1.76 (1.71, 1.81)**	**1.39 (1.35, 1.42)**
Continuous variables (β, 95% CI)				
Total t-score SD	**0.14 (0.13, 0.14)**	**0.12 (0.12, 0.13)**	**0.12 (0.11, 0.12)**	**0.05 (0.05, 0.06)**
DIMS t-score SD	**0.16 (0.15, 0.16)**	**0.14 (0.13, 0.14)**	**0.14 (0.13, 0.14)**	**0.07 (0.07, 0.08)**
SBD t-score SD	**0.13 (0.12, 0.13)**	**0.11 (0.11, 0.12)**	**0.11 (0.10, 0.12)**	**0.06 (0.06, 0.07)**
DA t-score SD	**0.14 (0.14, 0.15)**	**0.13 (0.12, 0.13)**	**0.13 (0.12, 0.13)**	**0.07 (0.06, 0.08)**
SWTD t-score SD	**0.13 (0.13, 0.14)**	**0.12 (0.11, 0.13)**	**0.12 (0.11, 0.12)**	**0.06 (0.05, 0.06)**
DOES t-score SD	**0.13 (0.13, 0.14)**	**0.12 (0.11, 0.13)**	**0.11 (0.11, 0.12)**	**0.06 (0.05, 0.06)**
SHY t-score SD	**0.18 (0.17, 0.18)**	**0.16 (0.15, 0.17)**	**0.16 (0.15, 0.16)**	**0.08 (0.07, 0.08)**

Model 1: adjusted for sex, age, BMI class, physical activity, low birth weight, premature birth, caesarean, breastfeeding, parental education, maternal age, insurance, family income class, second hand smoking exposure , petting ownership and $PM_{2.5}$ concentration. Effect estimates are expressed per one-standard-deviation increase in exposure. For continuous T-score outcomes, β coefficients represent the change in the outcome in standard deviation units per one-standard-deviation increase in exposure.

Table S11 Association between ∑CPs quantile levels and the risk of sleep disorder and subtypes among all participants in the logistic model.

Variables	Ref	Q2	Q3	Q4	*P for trend*
∑CP					
Sleep disorder	Ref.	**1.20 (1.11, 1.30)**	1.07 (0.98, 1.17)	**1.60 (1.48, 1.74)**	**<0.001**
DIMS	Ref.	**1.13 (1.06, 1.22)**	**1.12 (1.04, 1.20)**	**1.99 (1.86, 2.13)**	**<0.001**
SBD	Ref.	**1.63 (1.49, 1.78)**	**1.52 (1.39, 1.66)**	**1.50 (1.37, 1.64)**	**<0.001**
DA	Ref.	**1.12 (1.01, 1.25)**	**1.14 (1.03, 1.27)**	**2.36 (2.14, 2.60)**	**<0.001**
SWTD	Ref.	**1.62 (1.48, 1.78)**	**1.44 (1.31, 1.58)**	**1.73 (1.58, 1.90)**	**<0.001**
DOSE	Ref.	**1.45 (1.34, 1.58)**	**1.44 (1.32, 1.57)**	**2.14 (1.98, 2.32)**	**<0.001**
SHY	Ref.	**2.22 (2.05, 2.40)**	**2.22 (2.06, 2.40)**	**2.19 (2.02, 2.37)**	**<0.001**
Short sleep duration	Ref.	**12.05 (11.00, 13.21)**	**15.42 (14.02, 16.95)**	**95.32 (86.83, 104.64)**	**<0.001**
Long sleep platency	Ref.	**3.41 (2.93, 3.96)**	**3.79 (3.26, 4.42)**	**16.22 (14.03, 18.75)**	**<0.001**
∑SCCP					
Sleep disorder	Ref.	0.90 (0.82,0.98)	1.07 (0.98, 1.17)	**1.41 (1.30, 1.54)**	**<0.001**
DIMS	Ref.	0.92 (0.85, 0.99)	**1.08 (1.00, 1.17)**	**1.80 (1.68, 1.94)**	**<0.001**
SBD	Ref.	**1.10 (1.00, 1.20)**	**1.35 (1.24, 1.48)**	**1.29 (1.18, 1.41)**	**<0.001**
DA	Ref.	1.09 (0.98, 1.22)	**1.29 (1.16, 1.44)**	**2.02 (1.82, 2.24)**	**<0.001**
SWTD	Ref.	1.07 (0.97, 1.18)	**1.30 (1.18, 1.43)**	**1.46 (1.33, 1.61)**	**<0.001**
DOSE	Ref.	0.91 (0.83, 1.00)	**1.22 (1.11, 1.33)**	**1.68 (1.54, 1.82)**	**<0.001**
SHY	Ref.	**1.38 (1.28, 1.50)**	**1.54 (1.42, 1.67)**	**1.64 (1.51, 1.77)**	**<0.001**
Short sleep duration	Ref.	**1.70 (1.57, 1.83)**	**5.24 (4.89, 5.62)**	**22.04 (20.54, 23.66)**	**<0.001**
Long sleep platency	Ref.	**1.65 (1.43, 1.90)**	**2.90 (2.53, 3.32)**	**7.82 (6.86, 8.93)**	**<0.001**
∑MCCP					
Sleep disorder	Ref.	1.08 (0.99, 1.16)	0.98 (0.90, 1.06)	**1.35 (1.24, 1.46)**	**<0.001**
DIMS	Ref.	**1.10 (1.02, 1.17)**	1.04 (0.97, 1.12)	**1.72 (1.60, 1.84)**	**<0.001**
SBD	Ref.	**1.34 (1.23, 1.46)**	**1.37 (1.26, 1.49)**	**1.31 (1.20, 1.43)**	**<0.001**
DA	Ref.	1.03 (0.93, 1.14)	**1.12 (1.01, 1.24)**	**1.91 (1.73, 2.11)**	**<0.001**
SWTD	Ref.	**1.34 (1.22, 1.46)**	**1.30 (1.19, 1.42)**	**1.53 (1.39, 1.68)**	**<0.001**
DOSE	Ref.	**1.23 (1.14, 1.33)**	**1.23 (1.14, 1.34)**	**1.76 (1.62, 1.91)**	**<0.001**
SHY	Ref.	**1.41 (1.31, 1.52)**	**1.75 (1.63, 1.88)**	**1.67 (1.54, 1.80)**	**<0.001**
Short sleep duration	Ref.	**3.67 (3.44, 3.92)**	**4.42 (4.13, 4.72)**	**22.72 (21.27, 24.28)**	**<0.001**
Long sleep platency	Ref.	**1.76 (1.55, 1.99)**	**2.33 (2.06, 2.62)**	**7.33 (6.52, 8.24)**	**<0.001**
∑LCCP					
Sleep disorder	Ref.	1.03 (0.96, 1.11)	0.98 (0.91, 1.07)	1.05 (0.96, 1.14)	0.257
DIMS	Ref.	**1.16 (1.09, 1.24)**	**1.08 (1.01, 1.16)**	**1.25 (1.16, 1.34)**	**<0.001**
SBD	Ref.	1.03 (0.96, 1.12)	**1.09 (1.00, 1.19)**	1.05 (0.97, 1.15)	0.500
DA	Ref.	**1.14 (1.04, 1.25)**	**1.10 (1.00, 1.22)**	**1.35 (1.22, 1.49)**	**<0.001**
SWTD	Ref.	**1.12 (1.03, 1.21)**	**1.18 (1.07, 1.29)**	**1.25 (1.14, 1.37)**	**<0.001**

DOSE	Ref.	**1.12 (1.04, 1.20)**	**1.12 (1.04, 1.22)**	**1.21 (1.12, 1.32)**	**<0.001**
SHY	Ref.	**1.41 (1.32, 1.51)**	**1.40 (1.30, 1.52)**	**1.43 (1.33, 1.55)**	**<0.001**
Short sleep duration	Ref.	**3.73 (3.54, 3.93)**	**3.91 (3.68, 4.14)**	**5.60 (5.28, 5.94)**	**<0.001**
Long sleep platency	Ref.	**2.05 (1.86, 2.26)**	**1.91 (1.71, 2.13)**	**3.04 (2.73, 3.38)**	**<0.001**

Model 1: Adjusted for sex, age, BMI class, physical activity, low birth weight, premature birth, caesarean, breastfeeding, parental education, maternal age, insurance, family income class, second hand smoking exposure, petting ownership and $PM_{2.5}$ concentration

Bold: $P < 0.05$

Table S12 Association between ∑CPs quantile levels and the risk of sleep disorder and subtypes (t-score) among all participants in the logistic model.

Variables	Ref	Q2	Q3	Q4	*P for trend*
∑CP					
Total t-score	Ref.	**1.70 (1.53, 1.86)**	**1.73 (1.56, 1.90)**	**3.83 (3.66, 4.01)**	**<0.001**
DIMS t-score	Ref.	**1.99 (1.81, 2.17)**	**1.93 (1.75, 2.11)**	**4.58 (4.40, 4.77)**	**<0.001**
SBD t-score	Ref.	**1.52 (1.36, 1.68)**	**1.56 (1.40, 1.72)**	**3.44 (3.28, 3.60)**	**<0.001**
DA t-score	Ref.	**1.87 (1.72, 2.02)**	**1.88 (1.72, 2.03)**	**3.94 (3.78, 4.09)**	**<0.001**
SWTD t-score	Ref.	**1.68 (1.51, 1.84)**	**1.74 (1.57, 1.90)**	**3.82 (3.65, 3.99)**	**<0.001**
DOES t-score	Ref.	**1.60 (1.44, 1.76)**	**1.72 (1.56, 1.89)**	**3.62 (3.45, 3.79)**	**<0.001**
SHY t-score	Ref.	**2.77 (2.60, 2.93)**	**3.09 (2.92, 3.26)**	**5.43 (5.26, 5.61)**	**<0.001**
∑SCCP					
Total t-score	Ref.	0.11 (-0.07, 0.29)	**1.05 (0.87, 1.23)**	**2.92 (2.73, 3.10)**	**<0.001**
DIMS t-score	Ref.	**0.24 (0.05, 0.43)**	**1.16 (0.97, 1.36)**	**3.58 (3.39, 3.78)**	**<0.001**
SBD t-score	Ref.	**0.53 (0.36, 0.70)**	**1.31 (1.14, 1.49)**	**2.86 (2.68, 3.03)**	**<0.001**
DA t-score	Ref.	**0.64 (0.48, 0.81)**	**1.48 (1.32, 1.65)**	**3.13 (2.96, 3.30)**	**<0.001**
SWTD t-score	Ref.	**0.44 (0.27, 0.62)**	**1.40 (1.22, 1.58)**	**3.05 (2.87, 3.23)**	**<0.001**
DOES t-score	Ref.	-0.14 (-0.32, 0.03)	**0.90 (0.73, 1.08)**	**2.57 (2.39, 2.75)**	**<0.001**
SHY t-score	Ref.	**1.66 (1.48, 1.85)**	**2.43 (2.25, 2.61)**	**4.73 (4.55, 4.92)**	**<0.001**
∑MCCP					
Total t-score	Ref.	**0.81 (0.65, 0.97)**	**0.99 (0.83, 1.16)**	**2.83 (2.66, 3.01)**	**<0.001**
DIMS t-score	Ref.	**0.99 (0.82, 1.16)**	**1.27 (1.09, 1.44)**	**3.60 (3.41, 3.79)**	**<0.001**
SBD t-score	Ref.	**0.84 (0.69, 1.00)**	**1.10 (0.94, 1.26)**	**2.68 (2.51, 2.84)**	**<0.001**
DA t-score	Ref.	**0.93 (0.78, 1.07)**	**1.33 (1.18, 1.48)**	**3.01 (2.85, 3.18)**	**<0.001**
SWTD t-score	Ref.	**0.78 (0.62, 0.94)**	**1.14 (0.98, 1.30)**	**2.85 (2.68, 3.03)**	**<0.001**
DOES t-score	Ref.	**0.79 (0.63, 0.94)**	**0.87 (0.71, 1.03)**	**2.73 (2.55, 2.90)**	**<0.001**
SHY t-score	Ref.	**1.28 (1.12, 1.44)**	**2.22 (2.05, 2.38)**	**4.27 (4.09, 4.45)**	**<0.001**
∑LCCP					
Total t-score	Ref.	**0.56 (0.40, 0.71)**	**0.60 (0.42, 0.77)**	**1.07 (0.89, 1.25)**	**<0.001**
DIMS t-score	Ref.	**0.87 (0.71, 1.04)**	**1.00 (0.81, 1.19)**	**1.81 (1.62, 2.00)**	**<0.001**
SBD t-score	Ref.	**0.30 (0.15, 0.44)**	**0.62 (0.45, 0.78)**	**1.22 (1.05, 1.39)**	**<0.001**
DA t-score	Ref.	**0.80 (0.65, 0.94)**	**1.09 (0.93, 1.25)**	**1.68 (1.51, 1.84)**	**<0.001**
SWTD t-score	Ref.	**0.63 (0.48, 0.78)**	**0.72 (0.55, 0.89)**	**1.20 (1.02, 1.37)**	**<0.001**
DOES t-score	Ref.	**0.44 (0.29, 0.59)**	**0.67 (0.50, 0.84)**	**1.14 (0.96, 1.31)**	**<0.001**
SHY t-score	Ref.	**1.62 (1.46, 1.78)**	**1.81 (1.63, 1.99)**	**2.65 (2.47, 2.84)**	**<0.001**

Model 1: Adjusted for sex, age, BMI class, physical activity, low birth weight, premature birth, caesarean, breastfeeding, parental education, maternal age, insurance, family income class, second hand smoking exposure , petting ownership and $PM_{2.5}$ concentration

Bold: $P < 0.05$

Table S13 Association between ∑CPs exposures and the risk of sleep disorder and subtypes among participants without premature birth in the logistic model.

Variables	∑CPs	∑SCCPs	∑MCCPs	∑LCCPs
Categorical variables (OR, 95% CI)				
Sleep disorder	**1.19 (1.16, 1.23)**	**1.17 (1.14, 1.20)**	**1.14 (1.11, 1.17)**	**1.06 (1.04, 1.09)**
DIMS	**1.34 (1.31, 1.38)**	**1.29 (1.26, 1.32)**	**1.26 (1.23, 1.29)**	**1.11 (1.09, 1.13)**
SBD	**1.09 (1.05, 1.12)**	**1.08 (1.05, 1.11)**	**1.06 (1.03, 1.09)**	**1.03 (1.01, 1.05)**
DA	**1.35 (1.31, 1.39)**	**1.26 (1.22, 1.30)**	**1.28 (1.24, 1.32)**	**1.12 (1.10, 1.15)**
SWTD	**1.21 (1.17, 1.25)**	**1.17 (1.14, 1.21)**	**1.15 (1.12, 1.19)**	**1.08 (1.05, 1.10)**
DOSE	**1.29 (1.25, 1.33)**	**1.24 (1.21, 1.27)**	**1.22 (1.19, 1.26)**	**1.08 (1.06, 1.11)**
SHY	**1.16 (1.13, 1.19)**	**1.13 (1.11, 1.16)**	**1.13 (1.10, 1.16)**	**1.02 (1.01, 1.04)**
Short sleep duration	**3.34 (3.26, 3.42)**	**2.83 (2.77, 2.89)**	**2.32 (2.28, 2.37)**	**1.36 (1.34, 1.38)**
Long sleep platency	**1.87 (1.81, 1.92)**	**1.60 (1.56, 1.64)**	**1.72 (1.67, 1.77)**	**1.27 (1.24, 1.29)**
Continuous variables (β, 95% CI)				
Total t-score	**1.32 (1.25, 1.39)**	**1.15 (1.09, 1.21)**	**1.01 (0.95, 1.07)**	**1.01 (0.95, 1.07)**
DIMS t-score	**1.65 (1.58, 1.73)**	**1.41 (1.34, 1.47)**	**1.28 (1.22, 1.35)**	**1.28 (1.22, 1.35)**
SBD t-score	**1.25 (1.18, 1.31)**	**1.07 (1.02, 1.13)**	**0.95 (0.89, 1.00)**	**0.95 (0.89, 1.00)**
DA t-score	**1.36 (1.30, 1.42)**	**1.14 (1.09, 1.20)**	**1.07 (1.02, 1.12)**	**1.07 (1.02, 1.12)**
SWTD t-score	**1.30 (1.24, 1.37)**	**1.12 (1.06, 1.18)**	**1.01 (0.95, 1.07)**	**1.01 (0.95, 1.07)**
DOES t-score	**1.26 (1.20, 1.33)**	**1.10 (1.04, 1.16)**	**0.96 (0.90, 1.01)**	**0.96 (0.90, 1.01)**
SHY t-score	**1.92 (1.85, 1.99)**	**1.66 (1.60, 1.72)**	**1.49 (1.43, 1.55)**	**1.49 (1.43, 1.55)**

Model 1: exclude participants with premature birth, adjusted for sex, age, BMI class, physical activity, low birth weight, caesarean, breastfeeding, parental education, maternal age, insurance, family income class, second hand smoking exposure , petting ownership and $PM_{2.5}$ concentration

Bold: $P < 0.05$

Table S14 Association between ∑CPs exposures and the risk of sleep disorder and subtypes among participants without breastfeeding in the logistic model.

Variables	∑CPs	∑SCCPs	∑MCCPs	∑LCCPs
Categorical variables (OR, 95% CI)				
Sleep disorder	**1.18 (1.13, 1.23)**	**1.15 (1.11, 1.20)**	**1.14 (1.10, 1.18)**	**1.08 (1.04, 1.11)**
DIMS	**1.29 (1.24, 1.33)**	**1.25 (1.22, 1.29)**	**1.21 (1.17, 1.25)**	**1.11 (1.09, 1.14)**
SBD	**1.09 (1.05, 1.14)**	**1.07 (1.03, 1.11)**	**1.08 (1.04, 1.12)**	**1.05 (1.02, 1.08)**
DA	**1.34 (1.28, 1.40)**	**1.25 (1.20, 1.30)**	**1.29 (1.23, 1.34)**	**1.14 (1.10, 1.18)**
SWTD	**1.20 (1.15, 1.25)**	**1.16 (1.11, 1.21)**	**1.16 (1.11, 1.20)**	**1.08 (1.05, 1.12)**
DOSE	**1.29 (1.24, 1.34)**	**1.23 (1.19, 1.28)**	**1.24 (1.19, 1.29)**	**1.10 (1.07, 1.13)**
SHY	**1.14 (1.10, 1.19)**	**1.13 (1.09, 1.18)**	**1.11 (1.07, 1.15)**	**1.03 (1.00, 1.05)**
Short sleep duration	**3.08 (2.98, 3.18)**	**2.71 (2.63, 2.80)**	**2.31 (2.25, 2.37)**	**1.35 (1.32, 1.37)**
Long sleep platency	**1.81 (1.74, 1.89)**	**1.60 (1.54, 1.66)**	**1.67 (1.61, 1.74)**	**1.27 (1.23, 1.31)**
Continuous variables (β, 95% CI)				
Total t-score	**1.32 (1.22, 1.43)**	**1.16 (1.06, 1.26)**	**1.04 (0.95, 1.14)**	**0.48 (0.40, 0.55)**
DIMS t-score	**1.63 (1.52, 1.74)**	**1.42 (1.31, 1.52)**	**1.28 (1.18, 1.38)**	**0.63 (0.55, 0.71)**
SBD t-score	**1.31 (1.21, 1.41)**	**1.12 (1.02, 1.21)**	**1.04 (0.95, 1.13)**	**0.56 (0.49, 0.64)**
DA t-score	**1.32 (1.23, 1.42)**	**1.12 (1.03, 1.20)**	**1.06 (0.98, 1.15)**	**0.54 (0.47, 0.60)**
SWTD t-score	**1.31 (1.21, 1.41)**	**1.14 (1.05, 1.23)**	**1.04 (0.95, 1.13)**	**0.47 (0.40, 0.54)**
DOES t-score	**1.27 (1.18, 1.37)**	**1.11 (1.02, 1.20)**	**1.01 (0.92, 1.10)**	**0.45 (0.38, 0.52)**
SHY t-score	**1.92 (1.82, 2.03)**	**1.69 (1.59, 1.79)**	**1.52 (1.43, 1.61)**	**0.64 (0.57, 0.72)**

Model 1: exclude participants with breastfeeding, adjusted for sex, age, BMI class, physical activity, low birth weight, premature birth, caesarean, parental education, maternal age, insurance, family income class, second hand smoking exposure , petting ownership and $PM_{2.5}$ concentration

Bold: $P < 0.05$

Table S15 Association between ∑CPs exposures and the risk of sleep disorder and subtypes among participants without low birth weight in the logistic model.

Variables	∑CPs	∑SCCPs	∑MCCPs	∑LCCPs
Categorical variables (OR, 95% CI)				
Sleep disorder	**1.18 (1.14, 1.21)**	**1.15 (1.12, 1.18)**	**1.13 (1.10, 1.16)**	**1.07 (1.05, 1.09)**
DIMS	**1.32 (1.29, 1.35)**	**1.27 (1.24, 1.30)**	**1.24 (1.21, 1.27)**	**1.11 (1.09, 1.13)**
SBD	**1.08 (1.05, 1.11)**	**1.07 (1.04, 1.10)**	**1.06 (1.03, 1.09)**	**1.03 (1.01, 1.06)**
DA	**1.35 (1.30, 1.39)**	**1.26 (1.22, 1.30)**	**1.28 (1.24, 1.31)**	**1.13 (1.10, 1.15)**
SWTD	**1.18 (1.15, 1.22)**	**1.15 (1.12, 1.18)**	**1.14 (1.11, 1.17)**	**1.08 (1.05, 1.10)**
DOSE	**1.27 (1.23, 1.30)**	**1.22 (1.19, 1.25)**	**1.21 (1.18, 1.24)**	**1.09 (1.07, 1.11)**
SHY	**1.15 (1.12, 1.18)**	**1.12 (1.10, 1.15)**	**1.12 (1.10, 1.15)**	**1.02 (1.01, 1.04)**
Short sleep duration	**3.21 (3.14, 3.28)**	**2.79 (2.73, 2.85)**	**2.29 (2.25, 2.33)**	**1.36 (1.34, 1.37)**
Long sleep platency	**1.85 (1.80, 1.91)**	**1.60 (1.56, 1.64)**	**1.71 (1.66, 1.75)**	**1.28 (1.26, 1.31)**
Continuous variables (β, 95% CI)				
Total t-score	**1.32 (1.26, 1.39)**	**1.15 (1.09, 1.21)**	**1.02 (0.96, 1.08)**	**0.43 (0.38, 0.48)**
DIMS t-score	**1.66 (1.59, 1.73)**	**1.41 (1.35, 1.48)**	**1.29 (1.22, 1.35)**	**0.60 (0.55, 0.65)**
SBD t-score	**1.25 (1.19, 1.31)**	**1.07 (1.01, 1.13)**	**0.95 (0.90, 1.01)**	**0.48 (0.43, 0.52)**
DA t-score	**1.35 (1.29, 1.41)**	**1.14 (1.08, 1.19)**	**1.06 (1.01, 1.12)**	**0.49 (0.45, 0.53)**
SWTD t-score	**1.30 (1.23, 1.36)**	**1.12 (1.06, 1.18)**	**1.00 (0.95, 1.06)**	**0.43 (0.38, 0.47)**
DOES t-score	**1.27 (1.21, 1.34)**	**1.11 (1.05, 1.17)**	**0.97 (0.91, 1.03)**	**0.43 (0.38, 0.47)**
SHY t-score	**1.92 (1.85, 1.99)**	**1.66 (1.59, 1.72)**	**1.50 (1.44, 1.56)**	**0.60 (0.55, 0.65)**

Model 1: exclude participants with low birth weight, adjusted for sex, age, BMI class, physical activity, caesarean, premature birth, breastfeeding, parental education, maternal age, insurance, family income class, second hand smoking exposure , petting ownership and $PM_{2.5}$ concentration

Bold: $P < 0.05$
